# Newer antibiotics for drug-resistant Gram-negative infections in immunocompromised hosts: from pivotal trials to high-risk practice

**DOI:** 10.3389/fcimb.2026.1947603

**Published:** 2026-09-02

**Authors:** Yao Sun, Yiming Guo, Haizhen Cui, Yueyao Jiang, Qian Yu

**Affiliations:** 1 Department of Pharmacy, China-Japan Union Hospital of Jilin University, Changchun, China; 2 School of Pharmaceutical Sciences, Jilin University, Changchun, China

**Keywords:** antimicrobial resistance, drug-resistant Gram-negative bacteria, immunocompromised host, neutropenia, newer antibiotics, pharmacokinetics/pharmacodynamics, transplantation

## Abstract

Newer antibiotics have expanded treatment options for drug-resistant Gram-negative infections, but registration evidence is dominated by syndrome-based trials and post-approval evidence by heterogeneous observational cohorts. Translation to immunocompromised patients remains uncertain because profound neutropenia, transplantation, cell-targeted therapy, organ dysfunction, impaired source control, and limited immune-mediated clearance change both the probability and consequences of treatment failure. This critical narrative Review integrates 27 pivotal-trial protocol identifiers included in a predefined evidence map, immune-phenotype-specific cohorts, contemporary guidance, pharmacokinetic/pharmacodynamic evidence, and treatment-emergent resistance reports for a bounded core set of newer agents first authorized in the United States or European Union between 2014 and 2025, including newer β-lactam/β-lactamase-inhibitor combinations, cefiderocol, sulbactam–durlobactam, aztreonam–avibactam, and selected non-β-lactams. Fifteen of 27 pivotal protocols explicitly excluded at least one major immune phenotype or threshold; 11 had unresolved immune-host enrollment, and one enrolled immunocompromised patients but pooled distinct immune phenotypes. None reported comparative outcomes resolved to a defined immune phenotype. Phenotype-specific post-approval evidence was concentrated in hematological malignancy/profound neutropenia and solid-organ transplantation, whereas direct treatment-outcome evidence was sparse or absent for solid tumors, cellular therapies including CAR-T, advanced HIV infection, inborn errors of immunity/primary immunodeficiencies, and pediatric immunocompromised patients. We therefore separate phenotype-specific transportability from methodological credibility rather than treating directness as an overall certainty grade. The Review then synthesizes treatment by resistance mechanism and applies a host–pathogen–drug–context framework to empirical selection, diagnostics, exposure optimization, source control, de-escalation, relapse, and resistance. The resulting evidence map supports mechanism-active therapy while making the limits of transfer auditable and defining priorities for standardized phenotype reporting and pragmatic enrollment.

## 1 Introduction

Bacterial antimicrobial resistance (AMR) is already a major cause of preventable death rather than a distant threat. The Global Burden of Disease 2021 analysis estimated that 1.14 million deaths were attributable to bacterial AMR and 4.71 million deaths were associated with it in 2021. Among Gram-negative bacteria, carbapenem resistance increased more than resistance to any other antibiotic class between 1990 and 2021, reaching an estimated 216,000 attributable deaths in 2021 (Collaborators, 2024). The World Health Organization (WHO) Bacterial Priority Pathogens List consequently places particular emphasis on Gram-negative organisms resistant to last-resort agents and uses pathogen priority to guide antibacterial research, development, and public-health action (Organization, 2024). Its 2025 global surveillance report drew on more than 23 million bacteriologically confirmed infections reported from 104 countries in 2023, emphasizing both the scale of the problem and the need to interpret resistance through local epidemiology (Organization, 2025). These population-level estimates are not specific to immunocompromised patients, but they define the resistance landscape in which modern cancer therapy, transplantation, and immune-modifying treatment must operate.

Immunocompromise magnifies both exposure to resistant bacteria and the clinical cost of delayed active therapy. Recurrent health-care contact, invasive devices, mucosal barrier injury, surgery, repeated or prolonged antibiotic exposure, and colonization pressure intersect with defects in innate, cellular, or humoral immunity. Infection may present with attenuated or atypical inflammatory signs, while bacterial burden can increase rapidly and definitive source control may be difficult. Antibiotic exposure is therefore simultaneously more likely to be lifesaving and more likely to disrupt the microbiome, select resistance, and constrain future options (Liu et al., 2026). The label ‘immunocompromised,’ however, conceals substantial biological heterogeneity. A patient with prolonged profound neutropenia, a kidney-transplant recipient receiving stable maintenance immunosuppression, a person treated with a B-cell-depleting antibody, and a child with a primary immunodeficiency do not share the same barrier defects, pathogens, pharmacology, or capacity for immune-mediated bacterial clearance.

The resistance burden is especially visible in hematological malignancy and hematopoietic cell transplantation (HCT). A 2025 European Conference on Infections in Leukaemia (ECIL) systematic review included 40 studies, 33,387 patients or febrile episodes from observational studies, and 21,402 patients from one meta-analysis. Bloodstream infection prevalence averaged 30%; among Gram-negative bloodstream isolates, median resistance was 55% to fluoroquinolones, 30% for extended-spectrum β-lactamase production or third-generation cephalosporin resistance, and 13% for carbapenem resistance and multidrug resistance. Carbapenem resistance reached 26% in *Pseudomonas aeruginosa* and 38% in *Klebsiella pneumoniae*, with substantial geographical and temporal variation (Baccelli et al., 2025). These data do not justify universal use of the broadest regimen. They instead show why local epidemiology, prior colonization or infection, recent antibiotic exposure, clinical stability, and the expected depth and duration of immunosuppression must be integrated at the first treatment decision.

Therapeutic options have changed materially over the past decade. Ceftazidime–avibactam, ceftolozane–tazobactam, meropenem–vaborbactam, imipenem–cilastatin–relebactam, cefiderocol, sulbactam–durlobactam, aztreonam–avibactam, cefepime–enmetazobactam, plazomicin, and eravacycline provide activity against distinct combinations of resistant *Enterobacterales*, difficult-to-treat resistance (DTR) in *P. aeruginosa*, carbapenem-resistant *Acinetobacter baumannii* (CRAB), or metallo-β-lactamase (MBL) producers. Their spectra are not interchangeable, and activity depends on the resistance mechanism, infection site, susceptibility method and breakpoint, drug exposure, and organism-specific propensity for resistance emergence. Contemporary Infectious Diseases Society of America (IDSA) and European Society of Clinical Microbiology and Infectious Diseases (ESCMID) guidance therefore favors mechanism-informed selection and generally moves practice away from more toxic legacy regimens when an active newer agent is available (Paul et al., 2022; Tamma et al., 2024). The 10th European Conference on Infections in Leukaemia (ECIL-10) extends this principle to febrile neutropenia through a personalized empirical approach: broader coverage is prioritized for hemodynamic instability, previous resistant Gram-negative colonization or infection, or high-resistance settings, followed by de-escalation when resistant infection is excluded (Averbuch et al., 2026).

The central problem is not simply whether these agents work, but how confidently their pivotal evidence can be transferred to high-risk hosts. Registration programs are commonly structured around complicated urinary tract infection (cUTI), complicated intra-abdominal infection (cIAI), or hospital-acquired and ventilator-associated pneumonia, with noninferiority endpoints and eligibility criteria optimized for interpretable syndrome-level effects. Immune phenotype, intensity of immunosuppression, neutrophil recovery, concomitant immunomodulators, and transplant timing may be exclusion criteria, incompletely reported baseline characteristics, or absent subgroup variables. Across infectious-disease research, immunocompromised patients remain insufficiently studied; a recent multidisciplinary proposal for a dedicated trial network characterized the evidence deficit as a structural research-infrastructure problem (Hill et al., 2025). Even questions as fundamental as antibiotic duration have often been answered in populations that exclude immunocompromised patients, leaving clinicians to extrapolate from retrospective or heterogeneous data (Imlay et al., 2023).

Real-world studies partly fill this gap but introduce different uncertainties. Observational cohorts may preferentially include salvage therapy, mix colonization with infection, pool diverse immune defects, and compare agents used in different eras or at different levels of severity. Small samples, confounding by indication, survivor bias, inconsistent source control, variable susceptibility testing, and non-standard definitions of clinical success limit causal interpretation. At the same time, these cohorts capture precisely the conditions registration trials simplify: breakthrough infection during prophylaxis, polymicrobial disease, renal replacement therapy, extracorporeal support, interacting transplant medicines, persistent immunosuppression, recurrent bacteremia, and treatment-emergent resistance. A useful Review must therefore neither dismiss real-world evidence nor present it as equivalent to randomized evidence; it must specify which uncertainty each design resolves and which it leaves intact.

This Review critically evaluates newer antibiotics for drug-resistant Gram-negative infections in immunocompromised hosts. “Drug-resistant” is used as the umbrella term because extended-spectrum β-lactamase (ESBL) or AmpC production does not invariably satisfy a formal multidrug-resistant (MDR) definition. We ask four linked questions: who was represented in pivotal trials; what immune-phenotype-specific outcomes have been observed after approval; how host, pathogen, drug, and care-context factors should modify empirical and definitive therapy; and what study designs would close the most consequential gaps. The synthesis is organized by resistance mechanism and clinical decision rather than by drug alone.

## 2 Approach to evidence identification and interpretation

This article is a critical narrative Review. PubMed/MEDLINE searches were executed on 23 July 2026 and limited to publications from 1 January 2010 through 22 July 2026. The searches were supplemented by ClinicalTrials.gov records, regulatory and susceptibility-testing documents used to resolve eligibility or labeling, international guidance, and backward and forward citation tracking from pivotal trials and immune-host cohorts. Older sources were retained only when they defined resistance terminology, developmental pharmacology, or foundational principles. Complete Boolean strings, field restrictions, execution date, and non-deduplicated yields are provided in **Supplementary Table 1**: 288 records for the immune-host clinical query, 362 for the pivotal-trial query, and 1,147 for the pharmacokinetic/pharmacodynamic, resistance, and testing query. The streams overlap and are not additive.

For this Review, the structured core agent set was operationally bounded to systemic antibacterial agents or β-lactam/β-lactamase-inhibitor combinations first authorized in the United States or European Union between 1 January 2014 and 31 December 2025 and intended to address a clinically important resistant Gram-negative phenotype. Agent eligibility was assessed at the fixed regulatory cutoff of 31 December 2025, whereas literature concerning the included agents was updated through 22 July 2026. Products first authorized during 2026 and unapproved late-stage combinations were therefore outside the structured core map, even when subsequent development data became available during the evidence-update period. This bounded set was intended to support a reproducible evidence map rather than provide a complete inventory of the antibacterial development pipeline. Legacy agents used only as comparators and prophylactic strategies were also outside the core map.

The predefined core synthesis comprised 27 pivotal protocol identifiers (24 distinct current ClinicalTrials.gov records because three paired programs are now consolidated under a primary record and alias) and 11 immune-phenotype-specific treatment cohorts listed in **Supplementary Tables 2**, **3**. A clinically interpretable series required at least 10 treated patients, a defined antibacterial regimen, infection rather than colonization alone, a recognizable immune phenotype, and a clinical or microbiological outcome. The ≥10-patient threshold was a pragmatic mapping criterion rather than a validated methodological or quality cutoff. It was selected to distinguish cohort-level treatment evidence from isolated case reports and very small case series, in which individual outcomes can disproportionately influence percentage estimates and key determinants such as severity, source control, co-therapy, and immune recovery are difficult to characterize. All treatment cohorts identified through the database searches and backward and forward citation tracking that met these criteria were included in **Supplementary Table 3**. Reports involving fewer than 10 treated patients were not considered intrinsically uninformative; they were retained narratively when they addressed uncommon resistance mechanisms or distinctive pharmacological circumstances but were not used for corpus-level effectiveness statements. Prophylaxis-only studies, non-bacterial infections, and reports without interpretable treatment, phenotype, or outcome data were excluded from the core synthesis.

Evidence identification, eligibility assessment, and initial data extraction were performed by YS using the criteria described above. Database records were initially screened by YS; duplicate independent screening of the complete search sets was not undertaken. Included pivotal-trial entries were independently verified by YG and HC against the corresponding trial registry, primary publication, **Supplementary Material**, or regulatory source, and phenotype-specific cohort entries were independently verified by YJ. Verification included eligibility, immune phenotype, antibacterial exposure, outcome abstraction, representation state, and the methodological domains used for cohort appraisal. Verification covered eligibility, immune phenotype, antibacterial exposure, outcome abstraction, representation state, and the methodological domains used for cohort appraisal. Initial methodological-credibility placements for the phenotype-specific cohorts were assigned by YS and independently reassessed by YJ against the predefined domains and operational criteria. Differences in domain assessment, representation state, or credibility placement were discussed with YS and, when unresolved, adjudicated with QY until consensus was reached. The searches were supplemented by backward and forward citation tracking, and newly identified eligible studies were subjected to the same verification procedure. As this was a critical narrative Review, evidence identification combined source-specific database searches with backward and forward citation tracking, and the study-selection process is summarized in **Supplementary Figure 1**. No pooled treatment estimates were calculated; corpus-level numerical summaries are presented as descriptive counts within the predefined evidence maps.

Evidence was interpreted on two separate axes. Methodological credibility/internal validity concerns study design, treatment time-zero alignment, confounding, outcome ascertainment, statistical precision, and consistency. Phenotype-specific transportability concerns whether the immune defect and its trajectory, infection syndrome, resistance mechanism, severity, and treatment context correspond to the target patient. “Comparative” denotes a concurrent or explicitly time-aligned comparator in the same target population. “Well-adjusted” denotes a prespecified multivariable, matching, weighting, or propensity-based approach addressing, when available, baseline severity, infection source, time to active therapy, and source control. “Phenotype-specific” requires an operational immune definition and separately reported participant numbers or outcomes.

Placement along the methodological-credibility axis was descriptive and rule-based. Evidence was positioned toward lower methodological credibility when it was uncontrolled or when major limitations affected treatment time-zero alignment, control of confounding, or outcome ascertainment. Intermediate methodological credibility required a concurrent or explicitly time-aligned comparator or a well-adjusted observational design, together with reasonably defined treatment initiation and outcome ascertainment, while acknowledging residual confounding and imprecision. Higher methodological credibility was reserved for randomized comparisons or rigorous quasi-experimental designs with aligned treatment eligibility and time zero, appropriate comparator construction, prespecified outcome ascertainment, and no major methodological limitation that would materially undermine the treatment-effect estimate. Overall placement reflected study design and the extent to which major threats to internal validity were addressed. Phenotype-specific transportability was assessed separately according to representation state and the correspondence of immune phenotype, infection syndrome, resistance mechanism, severity, and treatment context to the target population.

Pivotal trials were abstracted for syndrome/pathogen eligibility, exact posted immune exclusions, corticosteroid and neutrophil thresholds, severity restrictions, eligibility state, demonstrable enrollment, phenotype outcomes, and source (**Supplementary Table 2**). Acute Physiology and Chronic Health Evaluation II (APACHE II) and Sequential Organ Failure Assessment (SOFA) scores are reported only where specified by the source. For each of the 11 phenotype-specific cohorts, methodological limitations were additionally appraised across structured domains comprising selection and treatment channeling, alignment of treatment time zero, control of measured confounding, reporting of source control, co-therapy and immune recovery, outcome ascertainment, and statistical precision (**Supplementary Table 5**). The structured domains in **Supplementary Table 5** informed placement along the methodological-credibility axis using the operational criteria above. Phenotype-specific transportability was assessed separately using representation state and phenotype/context correspondence.

Clinical recommendations were distinguished according to their evidentiary source. Statements were treated as direct clinical evidence when supported by pivotal trials or phenotype-specific treatment cohorts; as guideline-supported when derived from contemporary professional guidance; as PK/PD-supported when based primarily on pharmacological, modeling, or target-attainment evidence; and as expert interpretation when they represented a reasoned synthesis of resistance mechanism, host factors, and indirect evidence. Conditional language was retained when direct phenotype-specific comparative evidence was absent. Regulatory indications and age restrictions were considered separately from treatment recommendations and were based on jurisdiction-specific product information available at the evidence cutoff.

MDR, extensively drug-resistant, and pandrug-resistant follow internationally proposed acquired-resistance definitions where applicable (Magiorakos et al., 2012). DTR follows the treatment-option-centered construct of Kadri and colleagues; MDR and DTR are related but not interchangeable (Kadri et al., 2018).

## 3 Why immune phenotype changes the treatment question

The clinically relevant unit is not ‘immunocompromised versus immunocompetent,’ but the specific immune deficit, its intensity and trajectory, and the anatomic and treatment context in which infection occurs. Those variables influence the prior probability of resistance, how rapidly infection can progress, whether bactericidal exposure must compensate for impaired host clearance, the feasibility of source control, and the value placed on early broad therapy versus ecological restraint. **Table 1** summarizes a minimum phenotype description for interpreting antibacterial evidence; absolute neutrophil count (ANC) is standardized as cells/μL.

**Table 1:** Immune phenotypes and variables required for treatment interpretation.

Immune phenotype	Why bacterial treatment may differ	Minimum variables
Hematological malignancy/profound neutropenia/HCT	Mucosal barrier injury; quantitative innate immune deficit; central venous access; prophylactic and empirical antibiotic exposure	Depth and expected duration of neutropenia; mucositis; colonization; prior prophylaxis; hemodynamic stability; neutrophil recovery
Solid-organ transplantation	Surgical anatomy and devices plus chronic, changing immunosuppression; recurrent health-care exposure	Time from transplant; organ and anastomotic source; rejection treatment; renal/hepatic function; calcineurin-inhibitor exposure
Solid tumor receiving cytotoxic chemotherapy	Neutropenia and mucosal injury vary by regimen; devices, surgery, obstruction, and structural disease may dominate	Cancer type and treatment cycle; ANC trajectory; mucositis; device/source; recent surgery; corticosteroid and antibiotic exposure
Cell therapy and B-cell-targeted treatment	Cytopenias, hypogammaglobulinemia, mucosal injury, and cumulative prior therapy may overlap	Cellular product; timing; cytokine-directed therapy; steroid exposure; neutrophil and immunoglobulin trajectory
Autoimmune/inflammatory disease on intensive therapy	Variable cellular, humoral, and innate impairment; inflammatory signs may be suppressed	Specific biologic or small molecule; glucocorticoid dose/duration; combination immunosuppression; organ damage
Advanced HIV infection	Cell-mediated immune impairment with disease-stage and treatment-dependent bacterial risk	CD4 count; viral suppression; opportunistic coinfection; drug interactions; care access
Inborn error of immunity/primary immunodeficiency	Mechanistically diverse antibody, complement, phagocyte, or combined defects	Defined immune defect; replacement therapy; prior pathogens; anatomic sequelae; chronic colonization

Pediatric status is treated as a cross-cutting pharmacological and developmental modifier of every immune phenotype rather than as an immune phenotype itself; the phenotype-by-agent evidence-gap matrix is provided in **Supplementary Table 4**.

### 3.1 Hematological malignancy, neutropenia, and HCT

Profound neutropenia combines loss of a central effector of bacterial clearance with mucosal injury, vascular access, repeated hospitalization, and extensive prophylactic and empirical antibiotic exposure. The resulting infections are often bloodstream or pulmonary syndromes in which a few hours of inactive therapy may matter, yet fever may be the only early sign. Consistent with ECIL-10 guidance, empirical therapy should be individualized rather than based on a single universal regimen: hemodynamic instability, prior infection or colonization with a resistant Gram-negative organism, local resistance prevalence, recent antibiotics, and the expected duration of neutropenia inform whether initial coverage should include a newer mechanism-active agent (Averbuch et al., 2026). This strategy is guideline-derived, as randomized comparative evidence for empirical newer-agent use in defined immunocompromised phenotypes remains limited.

Immune recovery is also a competing determinant of outcome. In the TARZAN series of 54 carbapenemase-producing Enterobacterales bloodstream infections in neutropenic onco-hematology patients treated with ceftazidime–avibactam, 47% received inadequate initial empirical therapy; 7-day and 30-day case-fatality were 11% and 24%, and septic shock independently predicted 30-day mortality (Sastre-Escola et al., 2025). Other hematology cohorts report substantially higher mortality when pneumonia, mechanical ventilation, refractory malignancy, or prolonged profound neutropenia dominate (Halacoglu et al., 2025; Herrera et al., 2025). These studies support rapid active therapy but also warn against attributing every death or cure to the antibiotic alone. Similarly, a retrospective case–control study in acute leukemia after induction or HCT was strongly affected by treatment channeling and nonaligned treatment time zero, limiting comparative interpretation (Abi Frem et al., 2024). Neutrophil trajectory, source control, severity at treatment initiation, and adequacy of the empirical regimen must be reported alongside the definitive agent.

Solid-tumor patients receiving cytotoxic chemotherapy are within scope when neutropenia, mucosal injury, devices, obstruction, or recent surgery create a comparable treatment problem. However, none of the 11 core immune-host cohorts provided a phenotype-resolved newer-agent effect estimate specifically for this population; **Supplementary Table 4** therefore marks it as a major evidence gap rather than extrapolating hematology results without qualification.

### 3.2 Solid-organ transplantation

Solid-organ transplantation couples immune suppression to organ-specific anatomy. Early after transplant, surgical complications, drains, anastomoses, donor or recipient colonization, intensive-care exposure, and high immunosuppressive intensity shape infection. Later, urinary tract abnormalities in kidney recipients, biliary complications in liver recipients, and airway or allograft problems in lung recipients may sustain bacterial burden even when the systemic immune defect is less intense. The infection source and the feasibility of device removal or drainage can therefore matter as much as the nominal transplant status.

Post-transplant pharmacology constrains treatment selection. Renal dysfunction is common and can both reduce β-lactam clearance and magnify the toxicity of aminoglycosides or polymyxins; acute kidney injury may indirectly destabilize calcineurin-inhibitor exposure. In 81 kidney-transplant recipients treated with ceftazidime–avibactam for carbapenem-resistant Gram-negative infection, 30-day mortality was 22.2%, clinical cure 72.8%, and microbiological cure 66.7%; treatment within 48 hours was associated with cure, whereas APACHE II score predicted mortality (Zhang et al., 2024). A small liver-transplant comparison found broadly similar effectiveness for ceftazidime–avibactam and polymyxin B but more acute kidney injury with polymyxin B, illustrating why toxicity can favor a newer β-lactam even when comparative efficacy remains uncertain (Zhang et al., 2026). These cohorts have high transplant-phenotype transportability but low-to-moderate methodological credibility for a treatment effect; neither is a randomized transplant-specific estimate.

### 3.3 Cellular, humoral, and treatment-induced immunosuppression

Cellular therapies, B-cell-directed agents, targeted immunomodulators, and prolonged corticosteroids create overlapping rather than isolated defects. Chimeric antigen receptor T-cell (CAR-T) recipients may move through lymphodepletion, neutropenia, cytokine-release treatment, high-dose corticosteroid exposure, hypogammaglobulinemia, and recurrent hospitalization (Hill and Seo, 2020). In autoimmune or inflammatory disease, the same agent can confer different bacterial risk depending on dose, combination therapy, structural organ damage, and cumulative exposure. Because newer-antibiotic studies rarely report these variables, phenotype-specific treatment-outcome evidence is absent in the core map. The practical response is to document the time-varying immune phenotype, not to assume that a drug used successfully in HCT or solid-organ transplantation has been validated for cell therapy.

### 3.4 Advanced HIV infection and primary immunodeficiencies

Advanced HIV infection and inborn errors of immunity are especially poorly represented in registration programs. A low CD4 count changes the differential diagnosis, likelihood of coinfection, drug-interaction review, and competing mortality; viral suppression and immune recovery modify those risks, and current HIV guidance treats bacterial syndromes within this broader immune and treatment context (of the Infectious et al., 2025). Inborn errors of immunity span antibody, complement, phagocyte, combined, and immune-dysregulation phenotypes, often with chronic anatomic sequelae or colonization (Bousfiha et al., 2022). Neither population should be collapsed into a generic transplant category. The antibacterial mechanism remains relevant, but the evidence-gap matrix records no phenotype-resolved newer-agent treatment outcomes for either population.

### 3.5 Pediatric immunocompromised hosts

Pediatric evidence is limited by small numbers, weight-based dosing, formulation constraints, and sparse comparative and developmental safety data. Glomerular filtration and tubular function mature nonlinearly, while extracellular water and volume of distribution change substantially from the neonatal period through adolescence; adult dose scaling can therefore under- or over-expose a child even when the pharmacodynamic target is shared (Kearns et al., 2003). Critical illness, transplantation, nephrotoxic co-therapy, and rapidly changing renal function add further within-patient variability.

Adult efficacy may support regulatory or clinical extrapolation when infection biology and exposure targets are sufficiently similar, but it does not replace pediatric pharmacokinetic confirmation. Developmental safety and class-specific toxicity also matter: renal and auditory monitoring remains important for aminoglycoside-class agents such as plazomicin, whereas tetracycline-class effects on teeth and bone can restrict eravacycline use in younger patients according to jurisdiction-specific labeling. For a child with profound neutropenia, transplantation, or a primary immune defect, drug selection should pair mechanism-resolved susceptibility with current age-appropriate exposure guidance, formulation and organ-function review, and pediatric infectious-disease/pharmacy input; uncontrolled case series should not be interpreted as comparative effectiveness evidence.

Regulatory approval in pediatric patients remains agent-, syndrome-, age-, and jurisdiction-specific. At the evidence cutoff, ceftazidime–avibactam and ceftolozane–tazobactam had pediatric approvals in both the European Union and United States, although approved ages and pneumonia indications differed between jurisdictions; cefiderocol remained restricted to adults, with pediatric development ongoing (DailyMed, 2025; European Medicines Agency, 2025a; 2025b; DailyMed, 2026a; 2026b; European Medicines Agency, 2026). These regulatory distinctions inform prescribing but do not constitute comparative effectiveness evidence in immunocompromised children. Current jurisdiction-specific product information should therefore be consulted for age, dose, formulation, and indication restrictions.

Supporting programs included a randomized phase 2 cUTI trial of ceftazidime–avibactam and exposure-matching analyses for ceftolozane–tazobactam (Bradley et al., 2019; Larson et al., 2019). The PEDI-CEFI phase 2 study in 53 children aged 3 months to <18 years achieved concentrations above susceptibility breakpoints and reported no cefiderocol-related serious adverse events or discontinuations (Bradley et al., 2025). These developmental data support pediatric exposure and safety assessment but do not establish comparative effectiveness in profoundly immunocompromised children or justify use across all resistant pathogens and infection sites.

## 4 From eligibility to external validity: what pivotal trials tell us

Registration programs for newer Gram-negative agents used two main architectures. Syndrome-based trials established efficacy in cUTI, cIAI, or hospital-acquired bacterial pneumonia/ventilator-associated bacterial pneumonia (HABP/VABP) across multiple newer-agent programs (Solomkin et al., 2015; Wagenlehner et al., 2015; Mazuski et al., 2016; Wagenlehner et al., 2016; Kaye et al., 2018; Portsmouth et al., 2018; Torres et al., 2018; Kollef et al., 2019; Titov et al., 2021; Wunderink et al., 2021). Pathogen-directed programs increased microbiological relevance but generally used smaller, sometimes open-label comparisons (Carmeli et al., 2016; Wunderink et al., 2018; Motsch et al., 2020; Bassetti et al., 2021; Kaye et al., 2023; Carmeli et al., 2025).

**Supplementary Table 2** provides the complete trial-level evidence map of 27 pivotal protocol identifiers, corresponding to 24 distinct current registry records because three paired protocols are now consolidated under a primary record and an alias. Within this predefined map, 15 of 27 protocol identifiers (56%) explicitly excluded at least one major immune phenotype or threshold, 11 (41%) had unresolved immune-host enrollment, and one (4%) enrolled immunocompromised patients but reported them only as a heterogeneous pooled group. After removal of the three registry aliases, 14 of 24 distinct current records (58%) contained a broad or selective immune exclusion, nine of 24 (38%) had unresolved immune-host enrollment, and one of 24 (4%) had pooled immunocompromised representation. No protocol identifier or distinct registry record reported comparative outcomes resolved to a predefined immune phenotype.

Representation states were retained rather than collapsed into a binary included/excluded classification: broad exclusion (X-B), selective exclusion (X-S), eligible or not expressly excluded but enrollment unresolved (U), enrolled only within a heterogeneous pooled cohort (P), and phenotype-resolved enrollment and outcomes (R). Explicit exclusion confirms nonrepresentation; U leaves enrollment unknown; P confirms some representation but cannot support phenotype-specific inference. Similarly, allowance of limited corticosteroid exposure without subgroup reporting was not treated as evidence for biologically distinct states such as prolonged profound neutropenia.

Endpoint selection creates a second transportability problem. Many registration trials used noninferiority at a fixed test-of-cure visit, with syndrome-specific composites of clinical response and microbiological eradication. These endpoints are appropriate for registration but may not capture clinically important trajectories in high-risk hosts, including early inactive therapy, persistent or recurrent bacteremia, relapse after apparent cure, treatment-emergent resistance, toxicity that interrupts cancer or transplant therapy, and death before immune recovery. Follow-up may need to extend beyond the conventional test-of-cure window when immunosuppression and anatomic risk persist. The frequent exclusion of immunocompromised patients from antibiotic-duration studies illustrates the same limitation, because immune recovery can itself become a time-varying determinant of treatment success (Imlay et al., 2023).

**Figure 1:**
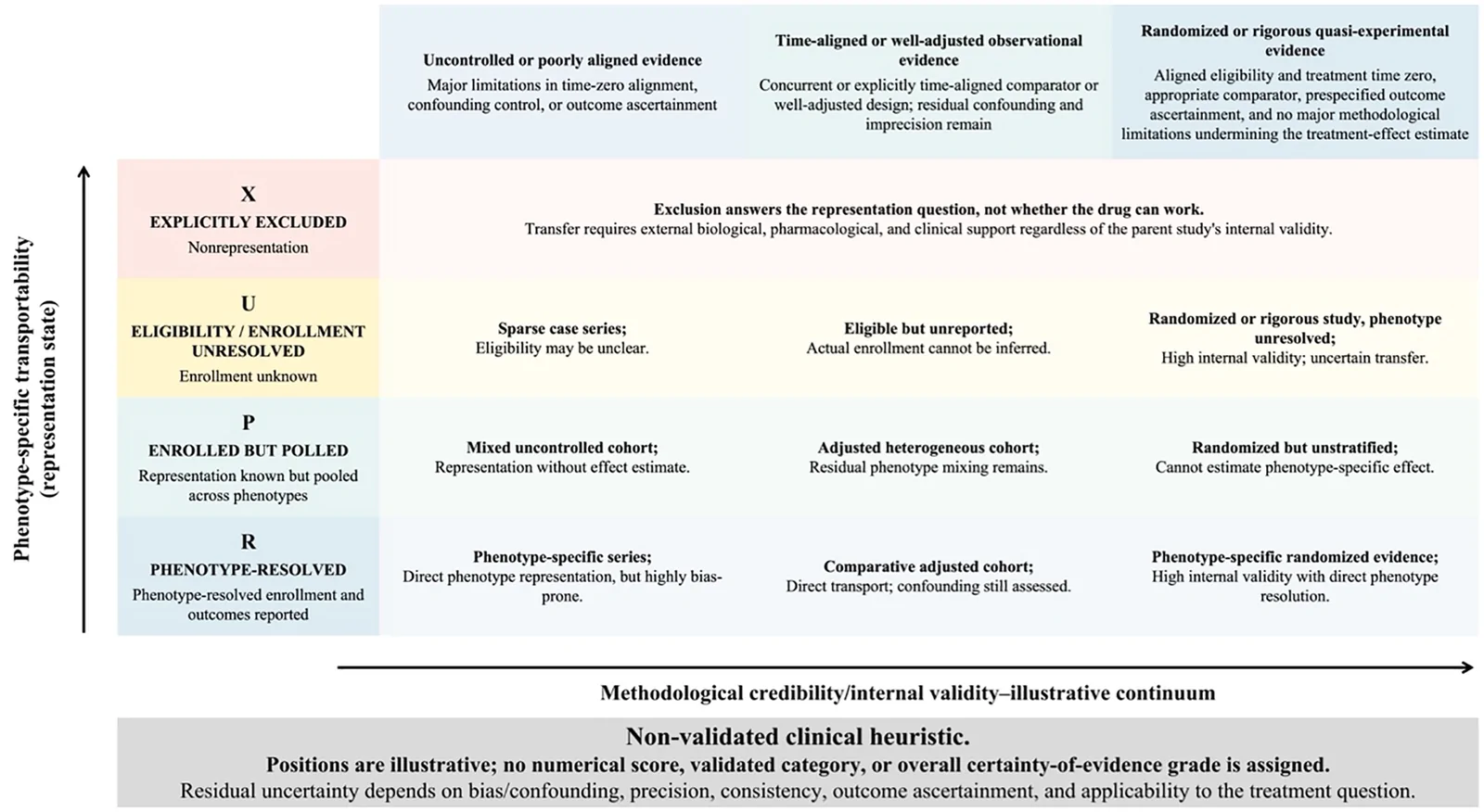
Conceptual map separating phenotype-specific transportability from methodological credibility. The vertical axis uses representation states X (explicitly excluded), U (eligible or not expressly excluded but enrollment unresolved), P (enrolled within a heterogeneous pooled cohort), and R (phenotype-resolved enrollment and outcomes). Representation state anchors the vertical position; full phenotype-specific transportability additionally depends on correspondence of the immune phenotype, infection syndrome, resistance mechanism, severity, and treatment context to the target population. The horizontal axis represents an illustrative methodological-credibility continuum based on study design, treatment time-zero alignment, control of confounding, outcome ascertainment, and statistical precision. The two axes are assessed separately. The framework is a non-validated clinical heuristic; positions are descriptive and do not constitute numerical scores, validated evidence categories, or an overall certainty-of-evidence grade.

**Figure 1** therefore replaces a one-dimensional directness ladder with two separate axes: phenotype-specific transportability and methodological credibility. A phenotype-specific observational series can have high phenotype-specific transportability yet low methodological credibility, whereas a rigorous randomized trial can have high methodological credibility but limited phenotype-specific transportability to an excluded phenotype. The matrix provides a descriptive clinical heuristic for these two dimensions, with methodological positions assigned using the operational criteria in Section 2.

## 5 Mechanism- and pathogen-directed treatment

A newer agent is not intrinsically ‘broader’ or ‘better’ across resistant Gram-negative bacteria. Its value depends on whether the resistance mechanism is within its spectrum, whether the infection syndrome supports adequate exposure, and whether using the agent improves safety or the probability of early activity without unnecessarily eroding future options. Phenotypic susceptibility remains essential, but rapid mechanism identification can prevent predictable mismatches—for example, using meropenem–vaborbactam against an OXA-48-like or metallo-β-lactamase producer. The following synthesis therefore begins with the organism and mechanism, then appraises phenotype-specific transportability and methodological credibility separately.

**Table 2** summarizes the principal mechanism-active options, available immune-host evidence, major evidence limitations, relevant infection sites, and practical PK/PD or safety considerations; the subsequent sections provide the supporting detail.

**Table 2:** Mechanism-oriented summary of newer-agent evidence and practical considerations in immunocompromised hosts.

Resistance mechanism/pathogen	Principal newer-agent options	Relevant infection sites/syndrome anchors	Available immune-host evidence	Principal limitations	Major PK/PD or safety considerations
ESBL- or AmpC-producing Enterobacterales	Established active β-lactams remain the usual benchmark when appropriate; cefepime–enmetazobactam, plazomicin, and eravacycline provide selected syndrome-specific alternatives according to susceptibility, infection site, and local guidance	cUTI/pyelonephritis for cefepime–enmetazobactam and plazomicin; cIAI for eravacycline	No core phenotype-specific comparative evidence supporting routine newer-agent use over established therapy in immunocompromised hosts	Pivotal programs frequently excluded major immune phenotypes; little direct evidence in profound neutropenia or transplantation	Preserve broader mechanism-active agents when narrower active therapy is adequate; plazomicin retains nephrotoxicity and ototoxicity concerns; eravacycline has gastrointestinal and hepatic tolerability considerations and lacks UTI/BSI efficacy evidence
KPC-producing or OXA-48-like CRE	Ceftazidime–avibactam; meropenem–vaborbactam; imipenem–cilastatin–relebactam for susceptible KPC producers; ceftazidime–avibactam also active against many OXA-48-like isolates	cUTI, cIAI, HAP/VAP and serious CRE infection including bacteremia, depending on agent and susceptibility	Ceftazidime–avibactam has the largest phenotype-specific experience, including hematological malignancy/neutropenia and kidney/liver transplantation; data for meropenem–vaborbactam and imipenem–relebactam are much less phenotype-specific	Predominantly observational evidence; confounding by indication, treatment timing, source control and immune recovery; no randomized phenotype-specific comparison	Renal dose adjustment and changing renal function are important; β-lactam accumulation can increase neurotoxicity risk; prior exposure or on-therapy failure should trigger repeat susceptibility/mechanism testing
MBL-producing Enterobacterales	Aztreonam–avibactam; synchronized aztreonam plus ceftazidime–avibactam when appropriate; cefiderocol if susceptible	Serious Gram-negative infection including bacteremia, cIAI and pneumonia according to agent/site-specific evidence	Limited mixed immunosuppressed cohorts support feasibility of mechanism-directed aztreonam protection or cefiderocol, but robust phenotype-specific comparative data are absent	REVISIT excluded profound neutropenia and selected transplant groups; ASSEMBLE was very small; immune phenotypes are pooled in most post-approval cohorts	When separate aztreonam and ceftazidime–avibactam are used, their administration should be coordinated to provide overlapping exposure; support for simultaneous administration and prolonged infusions derives mainly from PK/PD modeling; renal adjustment and confirmatory susceptibility testing are important
DTR *Pseudomonas aeruginosa*	Ceftolozane–tazobactam; ceftazidime–avibactam; imipenem–cilastatin–relebactam; cefiderocol according to susceptibility and mechanism	Pneumonia, bloodstream infection, cUTI and other serious infections according to agent	Relatively strong phenotype-specific immune-host evidence includes multicenter ceftolozane–tazobactam experience in immunocompromised patients and the ZENITH matched cohort in neutropenic hematology patients	Nonrandom treatment selection, calendar-era effects, residual severity/source-control confounding and limited phenotype-specific comparisons across agents	Pneumonia requires adequate pulmonary exposure; renal recovery or augmented clearance may reduce exposure; treatment-emergent resistance warrants repeat cultures and susceptibility testing
CRAB	Sulbactam–durlobactam in combination with imipenem–cilastatin or meropenem; cefiderocol when preferred options are inactive, unavailable, or unsuitable	HAP/VAP, bacteremia and other serious *A. baumannii* infections	ATTACK provides the strongest modern trial anchor but did not report immune-host subgroup outcomes; prospective cefiderocol experience includes pooled immunocompromised patients	Limited transportability of ATTACK to profoundly immunocompromised/unstable hosts; CREDIBLE-CR mortality imbalance and heterogeneous observational data complicate cefiderocol interpretation	Sulbactam–durlobactam was less nephrotoxic than colistin-based therapy; cefiderocol susceptibility testing requires particular laboratory care and unexpected/borderline results may require reference confirmation
*Stenotrophomonas maltophilia* and other difficult non-fermenters	For moderate-to-severe *S. maltophilia*, active combinations may include cefiderocol or ceftazidime–avibactam plus aztreonam alongside established non-β-lactam agents according to susceptibility and guidance	Respiratory, bloodstream and device-associated infection; distinction from colonization is essential	No robust phenotype-specific comparative treatment evidence; particularly relevant clinically after HCT, lung transplantation and prolonged critical-care exposure	Evidence derives largely from *in vitro*, PK/PD and nonrandomized clinical data; respiratory colonization can be misclassified as infection	Combination choice should be susceptibility- and mechanism-informed; toxicity and drug interactions of companion agents must be considered; microbiological confirmation is particularly important

BSI, bloodstream infection; cIAI, complicated intra-abdominal infection; CRE, carbapenem-resistant Enterobacterales; CRAB, carbapenem-resistant *Acinetobacter baumannii*; cUTI, complicated urinary tract infection; DTR, difficult-to-treat resistance; ESBL, extended-spectrum β-lactamase; HAP/VAP, hospital-acquired/ventilator-associated pneumonia; HCT, hematopoietic cell transplantation; KPC, *Klebsiella pneumoniae* carbapenemase; MBL, metallo-β-lactamase; PK/PD, pharmacokinetics/pharmacodynamics. The table is intended as a mechanism-oriented clinical summary rather than a treatment-ranking algorithm. Agent selection should remain conditional on susceptibility, infection site, current jurisdiction-specific guidance and labeling, organ function, prior antimicrobial exposure, and source control. Regulatory approval and recommended use may differ by jurisdiction; inclusion of an agent or infection site in this table does not imply regulatory approval for that indication.

Regulatory status and access also vary by jurisdiction. At the evidence cutoff, aztreonam–avibactam and cefepime–enmetazobactam had differing approved indications across the United States and European Union, while access to agents such as plazomicin was not uniform. Accordingly, every proposed use, age group, dose, and combination should be checked against the current local label and formulary rather than inferred from a trial or another jurisdiction.

### 5.1 ESBL-producing and AmpC-producing Enterobacterales

For severe infection caused by an ESBL-producing Enterobacterales isolate, a carbapenem remains the most established benchmark, particularly for bloodstream infection, pneumonia, critical illness, or a high-inoculum source. Ceftazidime–avibactam and other newer combinations may be active, but routine use for an otherwise carbapenem-susceptible ESBL isolate spends a mechanism-active option needed for carbapenem resistance without supplying immune-host-specific comparative benefit. Carbapenem-sparing decisions are most defensible in clinically stable patients with source control and a lower-inoculum urinary syndrome, interpreted against the full susceptibility profile and local guidance (Paul et al., 2022; Tamma et al., 2024). Profound neutropenia or instability lowers tolerance for an uncertain alternative; it does not make every newer agent preferable.

Cefepime–enmetazobactam demonstrated strong cUTI and acute-pyelonephritis efficacy in ALLIUM, but the trial excluded major immune-host categories and does not establish effectiveness for neutropenic bacteremia, pneumonia, or uncontrolled intra-abdominal infection (Kaye et al., 2022). For clinically significant AmpC-producing Enterobacterales, cefepime may be appropriate when susceptibility, dosing, infection site, and bacterial burden support it; a carbapenem is favored when those conditions are not met. Eravacycline has randomized evidence for cIAI but not for UTI or bloodstream infection, and its pivotal trials excluded immunocompromised patients (Solomkin et al., 2017, 2019). Plazomicin has cUTI evidence and a small carbapenem-resistant Enterobacterales signal, but nephrotoxicity, ototoxicity, availability, and limited immune-host representation constrain its role (McKinnell et al., 2019; Wagenlehner et al., 2019). Oral step-down is a syndrome- and guideline-specific strategy rather than a recommendation derived from the core immune-host evidence map. No core phenotype-specific cohort establishes a general oral step-down strategy during prolonged profound neutropenia.

### 5.2 KPC-producing and OXA-48-like carbapenem-resistant Enterobacterales

For KPC-producing Enterobacterales, ceftazidime–avibactam, meropenem–vaborbactam, and imipenem–cilastatin–relebactam are principal mechanism-active β-lactam options when the isolate is susceptible. Ceftazidime–avibactam additionally covers many OXA-48-like producers; vaborbactam and relebactam do not reliably solve OXA-48-like or metallo-β-lactamase resistance. TANGO II and RESTORE-IMI 1 supply credible pathogen-directed anchors, but their small samples and limited phenotype-resolved immune-host reporting constrain transportability to specific populations such as profound neutropenia and transplantation (Wunderink et al., 2018; Motsch et al., 2020). A general multicenter comparison found similar clinical success with meropenem–vaborbactam and ceftazidime–avibactam for carbapenem-resistant Enterobacterales, but it was observational and not immune-phenotype specific (Ackley et al., 2020). Selection therefore remains mechanism-, site-, susceptibility-, exposure-, and patient-specific rather than based on an assumed hierarchy among active agents.

Ceftazidime–avibactam has the largest phenotype-specific experience. In a 17-center cohort of 198 patients with hematological malignancy who received the drug for suspected or proven infection, 30-day mortality was 17.7%; septic shock and inappropriate initial therapy, rather than a simple immune-host label, independently predicted death (Tumbarello et al., 2025). TARZAN showed a 24% 30-day case-fatality rate in neutropenic carbapenemase-producing Enterobacterales bacteremia (Sastre-Escola et al., 2025). Among 41 acute-leukemia patients with carbapenemase-producing Enterobacterales bloodstream infection, overall 30-day mortality was 60.9%, while targeted ceftazidime–avibactam was associated with lower mortality after adjustment; the wide confidence interval and small sample require caution (Copaja-Corzo et al., 2025). A prospective cohort of 82 immunosuppressed patients receiving ceftazidime–avibactam or ceftazidime–avibactam plus aztreonam likewise showed high early response but worse outcomes with bacteremia, shock, and refractory malignancy (Herrera et al., 2025). These studies support feasibility and phenotype-specific clinical experience but do not establish randomized comparative effectiveness.

Interpretation of these cohorts remains limited by treatment channeling, heterogeneous treatment timing, source control, immune recovery, and residual confounding (**Supplementary Table 3** and **Supplementary Table 5**). Prior ceftazidime–avibactam exposure or on-therapy failure should prompt repeat susceptibility and mechanism testing because KPC variants, permeability changes, or mixed mechanisms may alter the preferred agent.

### 5.3 Metallo-β-lactamase-producing Enterobacterales

MBLs hydrolyze most β-lactams but not aztreonam. In clinical isolates, however, co-produced serine β-lactamases commonly inactivate aztreonam; avibactam protects it from those companion enzymes. This makes the fixed aztreonam–avibactam combination, or synchronized aztreonam plus ceftazidime–avibactam when the fixed product is unavailable, a mechanism-directed option. REVISIT compared aztreonam–avibactam with meropenem for serious Gram-negative infection, but it was descriptive, excluded profound neutropenia and selected transplant categories, and its dedicated MBL study ASSEMBLE enrolled only 15 patients (Carmeli et al., 2025). Cefiderocol is an alternative when susceptibility is confirmed and site-specific exposure is appropriate. Neither strategy has robust phenotype-specific comparative evidence in profound neutropenia or early transplantation.

The 82-patient immunosuppressed cohort reported by Herrera et al. included KPC and MBL producers treated with ceftazidime–avibactam alone or with aztreonam according to mechanism, while prospective cefiderocol cohorts have included MBL infections but generally pooled immune phenotypes and treatment indications (Giacobbe et al., 2024; Herrera et al., 2025). These data support feasibility rather than equivalence between regimens. When separate aztreonam and ceftazidime–avibactam products are used, PK/PD modeling supports coordinated administration to provide overlapping exposure. In a hollow-fiber model, simultaneous administration and prolonged infusion improved bacterial killing relative to staggered or shorter administration (Lodise et al., 2020). This constitutes PK/PD-model evidence rather than clinical-outcome proof; dosing, renal adjustment, infusion coordination, and local implementation should therefore follow agent-specific protocols and pharmacy review. Rapid carbapenemase identification and confirmatory susceptibility testing remain important when genotype and phenotype disagree.

### 5.4 Difficult-to-treat resistance in *Pseudomonas aeruginosa*

DTR *P. aeruginosa* is a treatment-option-centered phenotype rather than a single resistance mechanism; AmpC overexpression, porin loss, efflux, target changes, and acquired β-lactamases may coexist (Kadri et al., 2018; Tamma et al., 2024). Ceftolozane–tazobactam, ceftazidime–avibactam, imipenem–cilastatin–relebactam, and cefiderocol should therefore be selected according to susceptibility, resistance mechanism, infection site, and prior exposure rather than a fixed hierarchy. Ceftolozane–tazobactam has substantial pneumonia and post-approval experience, while ceftazidime–avibactam and imipenem–relebactam may be particularly relevant when the underlying β-lactamase mechanism is susceptible; cefiderocol provides an additional option but requires careful susceptibility interpretation.

The immune-host evidence for ceftolozane–tazobactam is comparatively informative but remains observational. A 14-center study of 69 immunocompromised patients with MDR *P. aeruginosa* reported 68% clinical cure and 19% 30-day mortality (Hart et al., 2021). In ZENITH, 44 neutropenic hematology patients with *P. aeruginosa* bloodstream infection treated with ceftolozane–tazobactam were matched to 88 controls; 7-day mortality was 6.8% versus 34.1% and 30-day mortality 22.7% versus 48.9%, with an adjusted association favoring ceftolozane–tazobactam (Bergas et al., 2022). Despite high phenotype-specific transportability, residual treatment selection and calendar-era confounding limit causal interpretation (**Supplementary Table 5**).

Treatment-emergent resistance remains clinically important when bacterial burden is high or source control and immune clearance are impaired. In an early 21-patient series, ceftolozane–tazobactam resistance emerged in three patients through AmpC-related mechanisms (Haidar et al., 2017). Persistent or recurrent infection should therefore prompt repeat culture and susceptibility testing rather than automatic continuation of the same agent. Pulmonary exposure and changing renal clearance are additional considerations in pneumonia.

### 5.5 Carbapenem-resistant *Acinetobacter baumannii*

CRAB is a distinct problem because active monotherapy is uncertain, pneumonia is frequent, and colonization can be difficult to separate from infection. ATTACK established sulbactam–durlobactam, administered with imipenem–cilastatin, as a less nephrotoxic non-inferior alternative to colistin plus imipenem–cilastatin for serious *A. baumannii*–*calcoaceticus* complex infection (Kaye et al., 2023). Contemporary guidance therefore anchors preferred CRAB therapy on sulbactam–durlobactam with a carbapenem; when unavailable, high-dose ampicillin–sulbactam within a combination regimen is an alternative (Tamma et al., 2024). Immune-host subgroup outcomes were not reported and sustained shock was excluded, limiting transportability to the most unstable immunocompromised hosts.

Cefiderocol requires a more guarded interpretation for CRAB. CREDIBLE-CR observed an imbalance in mortality in the cefiderocol arm, concentrated largely among *A. baumannii* infections, although the open-label descriptive design, heterogeneous best available therapy, and baseline imbalances complicate causality (Bassetti et al., 2021). In a prospective multicenter cohort of 185 cefiderocol-treated patients, 45% were immunocompromised; clinical cure among targeted infections was 42% for *A. baumannii* versus approximately 77–81% for Enterobacterales or *P. aeruginosa*, while immunocompromise itself was not independently associated with mortality (Lombardi et al., 2025). These findings support pathogen- and severity-specific caution rather than direct extrapolation from registration efficacy.

Cefiderocol susceptibility testing adds further uncertainty because routine methods may be sensitive to media, inoculum, and breakpoint-adjacent results, and standard minimum inhibitory concentration (MIC) testing may not identify resistant minority subpopulations (Simner et al., 2023; Irigoyen-von-Sierakowski et al., 2024). A small CRAB cohort also suggested clinically relevant heteroresistance, although the sample was insufficient for causal inference (Shields et al., 2024). Borderline, unexpected, or clinically discordant results should therefore prompt repeat testing and, where feasible, reference confirmation.

### 5.6 *Stenotrophomonas maltophilia* and other difficult non-fermenters

For *S. maltophilia*, the first task is to distinguish infection from respiratory or device colonization. This distinction is especially difficult after HCT, lung transplantation, or prolonged intensive-care exposure, where inflammatory signs may be muted and respiratory cultures repeatedly positive. Intrinsic L1 and L2 β-lactamases, efflux, and other mechanisms limit conventional β-lactams. Current guidance supports combination treatment for moderate-to-severe infection using two active agents, selected among trimethoprim–sulfamethoxazole, minocycline, levofloxacin, or cefiderocol, or ceftazidime–avibactam plus aztreonam in appropriate circumstances (Tamma et al., 2024). The supporting evidence is largely *in vitro*, PK/PD, or nonrandomized rather than phenotype-specific comparative evidence.

The same caution applies to less common non-fermenters such as *Burkholderia cepacia* complex or *Achromobacter* species. Species-level identification, repeat cultures, infection-site assessment, and specialized susceptibility testing are more informative than importing a regimen from *P. aeruginosa*. Because both phenotype-specific transportability and methodological credibility are generally limited, individualized treatment rationale is preferable to categorical drug recommendations.

## 6 Host-informed prescribing across pathogens

### 6.1 Empirical therapy, prior colonization, and rapid diagnostics

The empirical decision should separate the probability of resistance from the consequence of missing it. Hemodynamic instability, pneumonia, uncontrolled source, prolonged profound neutropenia, recent infection with the same resistant organism, and a local ecology with high resistance increase the expected harm of initially inactive therapy. Prior colonization is most actionable when recent, mechanism-resolved, and anatomically plausible; a remote rectal screen should not automatically dictate the broadest agent. ECIL-10 guidance supports early broadening in unstable or resistance-enriched febrile neutropenia and subsequent de-escalation when resistant infection is not confirmed (Averbuch et al., 2026). Direct comparative evidence for empirical newer-agent use in defined immune phenotypes remains limited. Accordingly, stable patients without these high-risk features may be managed with a narrower antipseudomonal β-lactam while diagnostics mature, according to syndrome, resistance risk, local epidemiology, and current guidance.

Rapid blood-culture identification and carbapenemase assays can shorten time to a mechanism-active agent, but they answer only part of the question. A resistance gene does not prove infection at another site; a negative panel does not exclude mechanisms outside its targets; and genotype does not replace phenotypic susceptibility, MIC interpretation, or source assessment. Results should be embedded in a stewardship pathway that can escalate, switch, or de-escalate immediately. Blood cultures before antibiotics, source cultures, review of prior isolates, and a clearly time-stamped empirical-to-definitive transition are essential for judging both care and evidence.

### 6.2 Pharmacokinetics/pharmacodynamics and optimized exposure

For β-lactams, maintaining adequate free-drug exposure relative to the MIC is a core PK/PD principle. PK/PD and target-attainment evidence supports prolonged infusion for selected time-dependent agents, but clinical-outcome evidence is mixed; prolonged or continuous infusion should therefore remain agent-, dose-, infection-site-, stability-, and protocol-specific rather than a universal consequence of critical illness or immunocompromise. Immunocompromise itself does not create a unique pharmacokinetic model; the associated physiology does. Augmented renal clearance, acute kidney injury, rapidly changing creatinine, fluid shifts, hypoalbuminemia, obesity, extracorporeal membrane oxygenation, and continuous or intermittent renal replacement therapy can all change exposure. Continuous ceftolozane–tazobactam infusion has been associated with a higher probability of target attainment in a real-world cohort, but clinical-outcome inference remains limited (Pilmis et al., 2019). Label dosing, contemporary renal-replacement guidance, and infection-site penetration should be reconciled rather than applying a single ‘immunocompromised dose.’

Therapeutic drug monitoring (TDM) is a conditional PK/PD-supported strategy rather than an established outcome-improving standard for newer β-lactams in immunocompromised hosts. Where available, it may be considered when exposure is especially uncertain, including rapidly changing clearance, extracorporeal support, a narrow therapeutic window, or an organism MIC near the breakpoint. Its value depends on a timely assay, a specified target, and the ability to change the regimen; an isolated concentration without these conditions adds false precision. Access remains a major limitation. Assays for newer β-lactams and inhibitor components commonly require locally validated chromatographic methods in specialist laboratories, turnaround may be too slow for an unstable patient, and harmonized exposure targets are incomplete. In a survey of US health systems with infectious-diseases pharmacy residencies, only 8% of respondents performed β-lactam TDM, with lack of rapid-turnaround testing the leading barrier (Chen et al., 2022). Where real-time concentrations cannot be obtained, a validated population-pharmacokinetic platform can combine dose history with body size, renal trajectory, continuous renal replacement therapy (CRRT) modality and effluent rate, and organism MIC to compare candidate regimens and probability of target attainment. Monte Carlo simulation is most useful for population or protocol design, whereas individualized Bayesian forecasting generally becomes more reliable after at least one measured concentration. *A priori* model-informed precision dosing (MIPD) can support, but not replace, clinical judgment or TDM: external model performance varies, creatinine can lag during acute kidney injury or augmented renal clearance, CRRT settings may change rapidly, and MIC itself is uncertain (Greppmair et al., 2023). Moreover, the multicenter DOLPHIN randomized trial of concentration-informed MIPD did not improve target attainment, intensive-care length of stay, or other clinical outcomes, so clinical benefit remains unproven (Ewoldt et al., 2022). Where neither TDM nor a locally validated model is available, serial renal function and urine output, dose and infusion audit, organism MIC and repeat cultures, clinical response, and toxicity remain pragmatic surrogates—not substitutes for a concentration, but actionable monitoring. In resource-limited settings, a minimum operational bundle is frequent renal-function and urine-output review, prompt maintenance-dose re-estimation as clearance changes, use of label- or guideline-based site-specific exposure rules, and early repeat culture when clinical and microbiological responses diverge. Loading-dose decisions should follow the agent-specific label, validated pharmacokinetic model, and local critical-care protocol; renal dysfunction commonly changes maintenance clearance, but it is not a universal instruction to retain or reduce every loading dose. Higher pharmacokinetic/pharmacodynamic targets at high-inoculum sites or during absent immune recovery remain a reasoned extrapolation rather than a proven outcome-improving strategy.

### 6.3 Concomitant medications, toxicity, and drug–drug interactions

In immunocompromised hosts, concomitant therapy encompasses both additional antimicrobial agents and background immunosuppressive, antineoplastic, antiviral, antifungal, and supportive-care regimens. These medications may modify antibacterial exposure, organ toxicity, immune recovery, and the feasibility of continuing cancer or transplant treatment and should therefore be documented when interpreting treatment outcomes.

A major benefit of newer β-lactams is the potential to avoid polymyxin- or aminoglycoside-dominant regimens. In TARZAN, reported nephrotoxicity occurred primarily among patients receiving concomitant aminoglycosides or colistin rather than being attributed to ceftazidime–avibactam itself (Sastre-Escola et al., 2025), while ATTACK demonstrated less nephrotoxicity with sulbactam–durlobactam than with colistin (Kaye et al., 2023). This does not mean that newer agents are toxicity-free. Accumulation may cause β-lactam neurotoxicity; aminoglycosides, including plazomicin, retain renal and auditory risks; and eravacycline may cause gastrointestinal and hepatic adverse effects. Frequent reassessment of renal and hepatic function is especially important during sepsis, transplant care, and periods of rapidly changing organ function.

Direct cytochrome-mediated interactions are generally fewer than those associated with azole antifungals or some antiretroviral agents, but indirect interactions remain clinically important. Antibiotic-associated kidney injury or renal recovery may alter calcineurin- or mTOR-inhibitor exposure; gastrointestinal intolerance may impair absorption of oral immunosuppressive or antiviral therapy; and additive marrow, renal, hepatic, or neurological toxicity may interrupt cancer treatment. Medication review should therefore encompass the full supportive-care regimen rather than the antimicrobial list alone. Changes in immunosuppression should be individualized in collaboration with the treating specialty; abrupt or complete withdrawal may threaten graft function and is not a substitute for active antibiotic therapy and source control.

### 6.4 Monotherapy, combination therapy, and source control

Empirical combination therapy and definitive combination therapy address different questions. As a probabilistic empirical strategy, use of two active-class candidates may increase the likelihood that at least one agent is active when shock, prior resistant colonization, or local epidemiology makes initial inactivity unacceptable; however, direct comparative evidence for this strategy in defined immune phenotypes is sparse. For definitive therapy, contemporary IDSA guidance generally does not support routine continuation of an additional nephrotoxic agent once a susceptible preferred β-lactam has been identified for several resistance phenotypes (Tamma et al., 2024). In the kidney-transplant ceftazidime–avibactam cohort, monotherapy and combination therapy did not differ significantly, but the observational design and sample size preclude an equivalence conclusion (Zhang et al., 2024). Mechanism- or guideline-defined exceptions include the carbapenem partner used with sulbactam–durlobactam for CRAB, avibactam protection of aztreonam against MBL-producing isolates when a fixed combination is unavailable, and combination strategies for moderate-to-severe *S. maltophilia* infection.

No antibiotic strategy compensates reliably for an undrained collection, infected obstructed system, retained removable catheter, devitalized tissue, or unstable surgical anastomosis. Source control may be more difficult in thrombocytopenia, early transplantation, or critical illness, but delay should be explicitly documented and revisited. Persistent bacteremia should prompt a source-control audit and repeat susceptibility before escalation to indefinite combination therapy. Intravascular devices, urinary obstruction, biliary anatomy, and ventilator-associated complications should be treated as clinically relevant determinants, not background comorbidities.

### 6.5 De-escalation, duration, and recurrence

De-escalation is an active safety intervention. When cultures and rapid diagnostics exclude the feared resistant pathogen, narrowing may be appropriate if the syndrome and clinical trajectory permit. As a guideline-derived recommendation, ECIL-10 permits discontinuation or narrowing in selected clinically stable neutropenic patients rather than automatically continuing broad therapy until neutrophil recovery (Averbuch et al., 2026). This recommendation should not be extrapolated as a universal stopping rule across immune phenotypes. Confidence decreases with persistent profound neutropenia, uncontrolled source, pneumonia, endovascular infection, or absent microbiological response. Direct immune-host evidence is insufficient to define a universal duration according to neutrophil recovery alone; duration should instead remain syndrome-specific and integrate source control, clinical stability, microbiological clearance, and immune trajectory (Imlay et al., 2023).

Follow-up must distinguish relapse from reinfection. Relapse with the same organism and mechanism suggests persistent source, inadequate exposure, or incomplete clearance; reinfection may reflect an enduring colonization reservoir and repeated health-care exposure. A plan for repeat cultures, catheter or anatomic reassessment, and future empirical therapy is particularly important after carbapenemase-producing Enterobacterales or DTR *P. aeruginosa*. Suppressive therapy is rarely a neutral solution: it can select resistance and disrupt the microbiome while postponing source control.

### 6.6 Treatment-emergent resistance and the colonization reservoir

Resistance can be present as a minority subpopulation at baseline or emerge through β-lactamase mutation, porin loss, efflux, target change, or altered iron transport during therapy. The clinical signal is most credible when serial isolates are linked by molecular typing, susceptibility methods are appropriate, drug exposure is documented, and the source is controlled. The ceftolozane–tazobactam resistance observed by Haidar and colleagues illustrates how quickly an initially active antipseudomonal β-lactam can be compromised (Haidar et al., 2017). For cefiderocol, TonB-dependent receptor mutations have been associated with heteroresistant *P. aeruginosa* populations, while population-analysis profiling, not routine MIC testing, is generally required to demonstrate the resistant subpopulation (Egge et al., 2024). Because this method is research-intensive and clinical outcome evidence remains preliminary, routine laboratories cannot reliably exclude heteroresistance. Treatment failure should therefore trigger repeat sampling, exposure and source-control review, and appropriately performed susceptibility testing; it should not be attributed to heteroresistance without supporting microbiology. A single susceptible baseline isolate is not a permanent property of the infection.

The intestinal and respiratory microbiome links the current episode to the next. Repeated prophylaxis and treatment can suppress susceptible flora, enrich resistant clones, and change the predictive value of prior screening. During high-dose chemotherapy or HCT, severe mucositis simultaneously disrupts the epithelial barrier and leaves a dense intestinal reservoir exposed to spatially heterogeneous antibiotic concentrations. In a pediatric HCT cohort, fecal strains were genetically indistinguishable from the subsequent blood isolate in all six evaluable mucosal-barrier-injury bloodstream infections, and increasing intestinal abundance was often temporally associated with antibiotic exposure (Kelly et al., 2019). It is therefore biologically plausible that high bacterial density and uneven luminal or mucosal exposure enrich pre-existing or newly selected minority variants—including porin-loss, AmpC-upregulated, or other β-lactamase variants—before failure is apparent in blood cultures. This sequence is a mechanistic inference rather than proven patient-level causation; gut drug exposure is agent-specific, and serial isolate sequencing plus exposure measurement would be required to demonstrate selection. Serial colonization data may help target future empirical therapy, but should not be used to treat asymptomatic carriage. Stewardship in immunocompromised hosts is thus longitudinal: record the mechanism and exposure history, minimize avoidable combination days, de-escalate when safe, and make the resistance consequence of today’s regimen visible to the next care team (Liu et al., 2026).

## 7 A host–pathogen–drug–context transfer framework

**Figure 2:**
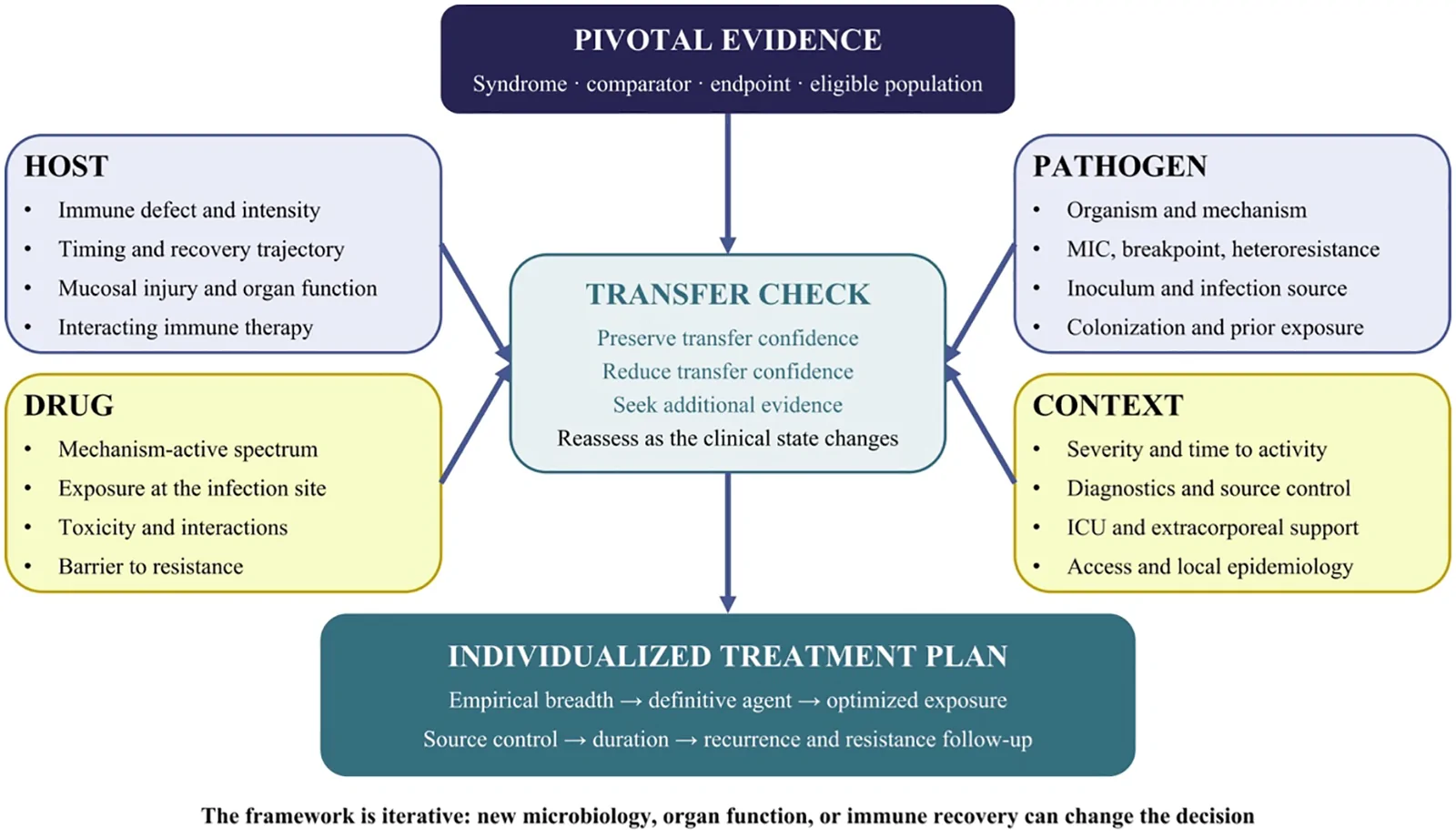
Host–pathogen–drug–context framework for transferring pivotal evidence to an individual immunocompromised host. Trial efficacy is the starting estimate. Immune phenotype and trajectory, mechanism-resolved microbiology, site-specific exposure and safety, and the care context determine whether transfer confidence is preserved or reduced and what safeguards are required. The framework is iterative and is intended as a clinical reasoning aid. ICU, intensive care unit; MIC, minimum inhibitory concentration.

The proposed framework treats pivotal efficacy as a starting estimate rather than a binary answer (**Figure 2**). The host domain asks which immune arm is impaired, how intensely, for how long, and with what organ dysfunction. The pathogen domain identifies the organism, resistance mechanism, inoculum, infection site, and relationship to prior colonization. The drug domain asks whether the agent is mechanism-active, can reach the target exposure, is tolerable with the full treatment regimen, and has a credible resistance barrier. The context domain captures variables that change faster than a guideline: hemodynamic stability, time to active therapy, diagnostic turnaround, source control, local epidemiology, critical-care support, and access.

These domains determine whether confidence in a trial estimate can be preserved, should be downgraded, or whether additional evidence is required. A stable kidney-transplant recipient with controlled-source cUTI, a susceptible isolate, and predictable renal function may remain close to a registration population. A patient with acute myeloid leukemia, profound neutropenia, pneumonia, shock, continuous renal replacement, and a borderline MIC does not. The latter difference does not prove the agent will fail; it increases uncertainty and justifies more aggressive diagnostics, optimized exposure, source control, and monitoring. Because immune recovery, organ function, and microbiology change, the transfer decision is iterative rather than a one-time score.

**Table 3** applies the framework to three deliberately contrasting cases. The examples illustrate a reasoning process, not patient-specific prescriptions; actual treatment remains contingent on current susceptibility, label, guideline, organ function, and multidisciplinary assessment.

**Table 3:** Worked applications of the evidence-transfer framework.

Clinical case	Evidence starting point	Transportability and credibility appraisal	Resulting safeguards
Controlled-source cUTI in a stable kidney-transplant recipient	Syndrome randomized-trial evidence plus transplant-specific ceftazidime–avibactam cohorts; some pivotal cUTI programs explicitly excluded renal transplant.	Transportability: moderate after accounting for stable graft function and source control. Credibility: high for syndrome efficacy, low-to-moderate for transplant comparative effect.	Confirm organism/mechanism and susceptibility; resolve obstruction/device; use agent- and jurisdiction-specific dosing; review renal trajectory and immunosuppressant toxicity; narrow when microbiology permits.
KPC bacteremia during prolonged profound neutropenia	Pathogen-directed evidence plus phenotype-specific ceftazidime–avibactam observational cohorts (including TARZAN); profound neutropenia was excluded or unresolved in several trials.	Transportability: limited by absent immune clearance, mucosal reservoir, and possible pneumonia/shock. Credibility: observational estimates remain vulnerable to timing and severity confounding.	Prioritize rapid mechanism-active therapy, time-stamp active treatment, optimize exposure using renal/MIC data, repeat blood cultures, audit source control, document neutrophil trajectory, and define relapse/resistance triggers.
CRAB pneumonia with shock and continuous renal replacement therapy	ATTACK supplies a randomized CRAB anchor; sustained shock was excluded and immune subgroups were not reported. Cefiderocol evidence is pathogen- and severity-sensitive.	Transportability: low for shock plus CRRT. Credibility: strong internal validity for the ATTACK population, but substantial phenotype/context gap for this case.	Distinguish infection from colonization; obtain reliable susceptibility testing; follow agent-specific guideline/label combination requirements; recalculate exposure with CRRT changes; repeat respiratory/blood cultures and reassess source control and resistance early.

**Figure 1** keeps two judgments visible. The vertical axis represents phenotype-specific transportability using the representation states X, U, P, and R, whereas the horizontal axis represents an illustrative continuum of methodological credibility based on the operational criteria in Section 2. The plotted positions are descriptive rather than numerical grades. Greater phenotype-specific transportability does not resolve confounding, and greater methodological credibility does not establish applicability to an excluded host. Each position should therefore be interpreted together with the residual uncertainty, monitoring plan, and trigger for reassessment.

## 8 Research priorities

The principal evidence gap is not a lack of additional uncontrolled cases; it is the absence of shared variables and comparative designs that make cases interpretable. Registration trials should replace broad routine exclusion with pragmatic enrollment where safety permits and prespecify immune-host strata. At minimum, studies should report the immune defect, intensity, timing and expected duration; neutrophil and lymphocyte trajectories where relevant; transplant type and time; recent rejection or cell-therapy treatment; corticosteroid exposure; organ function; and anticipated immune recovery. A binary ‘immunocompromised’ field is inadequate.

Create multicenter platforms across oncology, HCT, solid-organ transplantation, HIV, immunology, intensive care, microbiology, and antimicrobial stewardship so rare mechanism-defined infections can be enrolled prospectively.

Use prespecified immune-phenotype strata in registration and post-registration trials, with transparent counts for eligible, enrolled, analyzed, and lost participants.

Link outcomes to mechanism-resolved microbiology, standardized susceptibility methods, antibiotic exposure, source control, serial isolates, and longitudinal colonization data.

Design comparative-effectiveness studies that emulate a target trial, align time zero with treatment eligibility, and address confounding by indication, survivor bias, and time to active therapy.

Measure early mortality, persistent infection, clinical and microbiological failure, relapse and reinfection, organ toxicity, treatment-interrupting interactions, days alive outside hospital, and subsequent resistant infection.

Test exposure optimization and TDM in augmented clearance, rapidly changing renal function, renal replacement, extracorporeal support, and high-inoculum sites rather than assuming benefit from target attainment alone.

Embed rapid diagnostics and stewardship as interventions, measuring both time to active therapy and time to safe de-escalation.

A core outcome set and a minimum immune-phenotype dataset would make observational cohorts combinable without erasing biological differences. Prospective registries can then answer questions too rare for conventional trials, while nested randomization or platform methods evaluate empirical breadth, combination duration, infusion strategy, and stopping rules. The trial network proposed for infections in immunocompromised individuals provides a plausible infrastructure model (Hill et al., 2025). The goal is not to create a separate evidence universe for every immune defect, but to make the limits of transfer measurable and progressively smaller.

## 9 Discussion

The principal evidence gap is not the absence of active newer agents but uncertainty in transferring their efficacy to defined immunocompromised phenotypes. The pivotal evidence map demonstrates limited phenotype-resolved representation, whereas post-approval cohorts provide the strongest direct experience in neutropenic hematology and transplantation but remain vulnerable to treatment selection, treatment timing, source control, and immune recovery confounding. Consequently, phenotype specificity improves transportability but does not by itself establish comparative treatment effectiveness.

The most defensible clinical implications are conditional. Definitive selection should align resistance mechanism, susceptibility, infection site, current jurisdiction-specific guidance and labeling, and patient-level treatment constraints. Empirical breadth depends on instability, prior isolates, local ecology, and the consequences of initial inactivity, while narrowing or discontinuation remains syndrome- and trajectory-specific. Dose and infusion decisions should follow agent-specific evidence as renal function and extracorporeal support change. Combination therapy, oral step-down, and treatment before immune recovery should therefore remain guideline-, syndrome-, or mechanism-specific strategies rather than universal consequences of immunocompromise. Accordingly, direct phenotype-specific clinical evidence, guideline-derived recommendations, PK/PD-supported strategies, and expert interpretation are identified separately throughout this Review rather than treated as equivalent sources of support.

The greatest uncertainty persists where mechanistic plausibility exceeds phenotype-specific comparative evidence. For MBL-producing Enterobacterales, aztreonam protected by avibactam and cefiderocol are mechanistically credible but phenotype-specific comparative data are sparse. For CRAB, sulbactam–durlobactam provides the strongest modern trial anchor, whereas cefiderocol must be interpreted in light of the CREDIBLE-CR mortality imbalance and mixed real-world outcomes. For *S. maltophilia* and other difficult non-fermenters, distinguishing infection from colonization, obtaining reliable species-level microbiology, and interpreting limited nonrandomized evidence may be as important as drug selection itself. In these settings, the framework is intended to make uncertainty explicit rather than convert mechanistic activity into an unsupported categorical recommendation.

Access and laboratory capacity also constrain applicability. Carbapenemase testing, reference susceptibility methods, therapeutic drug monitoring, and several newer agents are not uniformly available, while breakpoints, approved indications, and recommended uses can differ by jurisdiction. A theoretically preferred regimen that cannot be delivered promptly is not necessarily superior to an active, safely administered alternative. Local antibiograms should therefore be interpreted, where possible, alongside immune-host and unit-specific resistance patterns because oncology, transplant, and intensive-care ecologies may differ substantially from hospital-wide estimates.

This critical narrative Review has several limitations. Registry information may not capture site-level enrollment decisions, and absence of subgroup reporting does not establish absence of enrollment. Observational studies remain heterogeneous in treatment time zero, phenotype definition, infection source, outcome ascertainment, and adjustment. The structured appraisal of phenotype-specific cohorts (**Supplementary Table 5**) and the two-axis framework were therefore used to distinguish methodological credibility from phenotype-specific transportability. Evidence transfer should identify the phenotype-specific gap, use the most relevant mechanism- and syndrome-based anchor, incorporate observational evidence with appropriate attention to residual uncertainty, and allow reassessment as microbiology, organ function, source control, and immune recovery change.

## 10 Conclusion

Newer mechanism-active agents expand treatment options for drug-resistant Gram-negative infection, but evidence across defined immunocompromised phenotypes remains markedly uneven. Phenotype-specific clinical experience is concentrated in hematological malignancy/profound neutropenia and solid-organ transplantation, whereas solid tumors, cellular therapies including CAR-T, advanced HIV infection, inborn errors of immunity/primary immunodeficiencies, and pediatric immunocompromised patients remain major evidence gaps. Transfer from pivotal trials should therefore be judged separately for methodological credibility and phenotype-specific transportability, then conditioned on resistance mechanism, infection site, exposure, toxicity, source control, and immune trajectory. Trial-level eligibility reporting, pragmatic enrollment, standardized immune-phenotype datasets, prospective comparisons, and serial microbiology are needed to make high-risk practice more reliable.
