# Supplementary material for: Newer antibiotics for drug-resistant Gram-negative infections in immunocompromised hosts: from pivotal trials to high-risk practice
Source: Front Cell Infect Microbiol. 2026 Sep 2;16:1947603. doi: 10.3389/fcimb.2026.1947603 (PMC13579259; doi:10.3389/fcimb.2026.1947603)
Supplement: Supplementary file 1 [file SupplementaryFile1.docx]

Supplementary Figure S1. Evidence identification and classification pathway

Supplementary Table S1. Reproducible evidence-identification approach

Design clarification

This is a critical narrative Review, not a systematic review or meta-analysis. Database discovery yields are reported for reproducibility, but the streams overlap and were supplemented by registry reconstruction, regulatory sources, and citation chaining. They therefore do not form a deduplicated closed sample suitable for a PRISMA flow diagram.

| **Evidence stream** | **Source** | **Execution date** | **Publication/record window** | **Search structure** | **Yield** |
| --- | --- | --- | --- | --- | --- |
| Immune-host clinical evidence | PubMed/MEDLINE | 23 Jul 2026 | 1 Jan 2010–22 Jul 2026 | Immune-host block AND resistance/organism block AND agent block | 288 |
| Pivotal-trial evidence | PubMed/MEDLINE | 23 Jul 2026 | 1 Jan 2010–22 Jul 2026 | Resistance/organism block AND agent block AND trial-design block | 362 |
| PK/PD, resistance, and testing evidence | PubMed/MEDLINE | 23 Jul 2026 | 1 Jan 2010–22 Jul 2026 | Resistance/organism block AND agent block AND support block | 1,147 |
| Pivotal registry map | ClinicalTrials.gov | 23 Jul 2026 | Records available through 22 Jul 2026 | Generic drug names, development codes, named trials, and every protocol identifier in Table S2 | 27 protocol identifiers / 24 current records |
| Supplementary sources | Regulatory/standards/guidance and citation tracking | Iterative through 23 Jul 2026 | Current versions where relevant | Agent + label/assessment/AST; WHO/IDSA/ESCMID/ECIL; backward/forward citations from pivotal trials and immune-host cohorts | Purposive; no additive yield |

Search 1: immune-host clinical evidence (288 records)

(
"Immunocompromised Host"[Mesh] OR immunocompromis*[Title/Abstract] OR
neutropeni*[Title/Abstract] OR "hematologic malignan*"[Title/Abstract] OR
"hematological malignan*"[Title/Abstract] OR leukemia[Title/Abstract] OR
lymphoma[Title/Abstract] OR "hematopoietic cell transplant*"[Title/Abstract] OR
"hematopoietic stem cell transplant*"[Title/Abstract] OR HCT[Title/Abstract] OR
HSCT[Title/Abstract] OR "solid organ transplant*"[Title/Abstract] OR
"kidney transplant*"[Title/Abstract] OR "liver transplant*"[Title/Abstract] OR
"CAR-T"[Title/Abstract] OR "chimeric antigen receptor"[Title/Abstract] OR
"B-cell deplet*"[Title/Abstract] OR rituximab[Title/Abstract] OR
"advanced HIV"[Title/Abstract] OR AIDS[Title/Abstract] OR
"primary immunodeficien*"[Title/Abstract] OR
"inborn errors of immunity"[Title/Abstract] OR
immunosuppress*[Title/Abstract] OR corticosteroid*[Title/Abstract]
)
AND
(
(
"Gram-Negative Bacterial Infections"[Mesh] OR "Gram-negative"[Title/Abstract] OR
Enterobacterales[Title/Abstract] OR Enterobacteriaceae[Title/Abstract] OR
"Pseudomonas aeruginosa"[Title/Abstract] OR
"Acinetobacter baumannii"[Title/Abstract] OR
"Stenotrophomonas maltophilia"[Title/Abstract]
) AND (
"Drug Resistance, Bacterial"[Mesh] OR resist*[Title/Abstract] OR
ESBL[Title/Abstract] OR "extended-spectrum beta-lactamase"[Title/Abstract] OR
AmpC[Title/Abstract] OR carbapenemase*[Title/Abstract] OR
"metallo-beta-lactamase"[Title/Abstract] OR
"difficult-to-treat"[Title/Abstract] OR MDR[Title/Abstract] OR XDR[Title/Abstract]
)
)
AND
(
"ceftazidime-avibactam"[Title/Abstract] OR
"ceftolozane-tazobactam"[Title/Abstract] OR
"meropenem-vaborbactam"[Title/Abstract] OR
"imipenem-cilastatin-relebactam"[Title/Abstract] OR
"imipenem-relebactam"[Title/Abstract] OR cefiderocol[Title/Abstract] OR
"sulbactam-durlobactam"[Title/Abstract] OR
"aztreonam-avibactam"[Title/Abstract] OR
(aztreonam[Title/Abstract] AND "ceftazidime-avibactam"[Title/Abstract]) OR
"cefepime-enmetazobactam"[Title/Abstract] OR plazomicin[Title/Abstract] OR
eravacycline[Title/Abstract]
)
AND
("2010/01/01"[Date - Publication] : "2026/07/22"[Date - Publication])

Search 2: pivotal-trial evidence (362 records)

(
(
"Gram-Negative Bacterial Infections"[Mesh] OR "Gram-negative"[Title/Abstract] OR
Enterobacterales[Title/Abstract] OR Enterobacteriaceae[Title/Abstract] OR
"Pseudomonas aeruginosa"[Title/Abstract] OR
"Acinetobacter baumannii"[Title/Abstract] OR
"Stenotrophomonas maltophilia"[Title/Abstract]
) AND (
"Drug Resistance, Bacterial"[Mesh] OR resist*[Title/Abstract] OR
ESBL[Title/Abstract] OR "extended-spectrum beta-lactamase"[Title/Abstract] OR
AmpC[Title/Abstract] OR carbapenemase*[Title/Abstract] OR
"metallo-beta-lactamase"[Title/Abstract] OR
"difficult-to-treat"[Title/Abstract] OR MDR[Title/Abstract] OR XDR[Title/Abstract]
)
)
AND
(
"ceftazidime-avibactam"[Title/Abstract] OR
"ceftolozane-tazobactam"[Title/Abstract] OR
"meropenem-vaborbactam"[Title/Abstract] OR
"imipenem-cilastatin-relebactam"[Title/Abstract] OR
"imipenem-relebactam"[Title/Abstract] OR cefiderocol[Title/Abstract] OR
"sulbactam-durlobactam"[Title/Abstract] OR
"aztreonam-avibactam"[Title/Abstract] OR
(aztreonam[Title/Abstract] AND "ceftazidime-avibactam"[Title/Abstract]) OR
"cefepime-enmetazobactam"[Title/Abstract] OR plazomicin[Title/Abstract] OR
eravacycline[Title/Abstract]
)
AND
(
randomized controlled trial[Publication Type] OR random*[Title/Abstract] OR
"phase 3"[Title/Abstract] OR "phase III"[Title/Abstract] OR pivotal[Title/Abstract]
)
AND
("2010/01/01"[Date - Publication] : "2026/07/22"[Date - Publication])

Search 3: PK/PD, resistance, and testing evidence (1,147 records)

(
(
"Gram-Negative Bacterial Infections"[Mesh] OR "Gram-negative"[Title/Abstract] OR
Enterobacterales[Title/Abstract] OR Enterobacteriaceae[Title/Abstract] OR
"Pseudomonas aeruginosa"[Title/Abstract] OR
"Acinetobacter baumannii"[Title/Abstract] OR
"Stenotrophomonas maltophilia"[Title/Abstract]
) AND (
"Drug Resistance, Bacterial"[Mesh] OR resist*[Title/Abstract] OR
ESBL[Title/Abstract] OR "extended-spectrum beta-lactamase"[Title/Abstract] OR
AmpC[Title/Abstract] OR carbapenemase*[Title/Abstract] OR
"metallo-beta-lactamase"[Title/Abstract] OR
"difficult-to-treat"[Title/Abstract] OR MDR[Title/Abstract] OR XDR[Title/Abstract]
)
)
AND
(
"ceftazidime-avibactam"[Title/Abstract] OR
"ceftolozane-tazobactam"[Title/Abstract] OR
"meropenem-vaborbactam"[Title/Abstract] OR
"imipenem-cilastatin-relebactam"[Title/Abstract] OR
"imipenem-relebactam"[Title/Abstract] OR cefiderocol[Title/Abstract] OR
"sulbactam-durlobactam"[Title/Abstract] OR
"aztreonam-avibactam"[Title/Abstract] OR
(aztreonam[Title/Abstract] AND "ceftazidime-avibactam"[Title/Abstract]) OR
"cefepime-enmetazobactam"[Title/Abstract] OR plazomicin[Title/Abstract] OR
eravacycline[Title/Abstract]
)
AND
(
"Pharmacokinetics"[Mesh] OR pharmacokinetic*[Title/Abstract] OR
pharmacodynamic*[Title/Abstract] OR "therapeutic drug monitoring"[Title/Abstract] OR
"model-informed precision dosing"[Title/Abstract] OR MIPD[Title/Abstract] OR
"resistance emergence"[Title/Abstract] OR
"treatment-emergent resistance"[Title/Abstract] OR heteroresistance[Title/Abstract] OR
"susceptibility testing"[Title/Abstract]
)
AND
("2010/01/01"[Date - Publication] : "2026/07/22"[Date - Publication])

Definitions, selection, and verification

Core newer-agent set: systemic antibacterial agents or β-lactam/β-lactamase-inhibitor combinations first authorized in the United States or European Union between 1 January 2014 and 31 December 2025 and intended to address a clinically important resistant Gram-negative phenotype. Agent eligibility was assessed at the fixed regulatory cutoff of 31 December 2025; evidence concerning the included agents was updated through 22 July 2026. Products first authorized during 2026 and unapproved late-stage combinations were outside the structured core map.

Core synthesis: the 27 pivotal protocol identifiers in Table S2 and 11 immune-phenotype-specific treatment cohorts in Table S3.

Clinically interpretable series: at least 10 treated patients, a defined antibacterial regimen, infection rather than colonization alone, a recognizable immune phenotype, and a clinical or microbiological outcome. All treatment cohorts identified through the database searches and backward and forward citation tracking that met these criteria were included in Table S3.

YS performed initial evidence identification, eligibility assessment, and data extraction. To strengthen the reproducibility of the structured evidence maps, YG and HC independently rechecked all included pivotal-trial entries against the linked registry, primary publication, supplement, or regulatory source, and YJ independently rechecked all included phenotype-specific cohort entries. Verification covered eligibility, immune phenotype, antibacterial exposure, outcome abstraction, representation state, and the methodological domains used for cohort appraisal. Initial methodological-credibility placements for the phenotype-specific cohorts were assigned by YS and independently reassessed by YJ against the predefined domains and operational criteria. Differences in domain assessment, representation state, or credibility placement were discussed with YS and, when unresolved, adjudicated with QY until consensus was reached.

Prophylaxis-only studies, non-bacterial infections, and reports without interpretable treatment, phenotype, or outcome data were excluded from the core synthesis.

No registered protocol, duplicate independent screening of the complete discovery sets, formal validated risk-of-bias instrument, or pooled treatment estimate was used.

Representation states: X-B, broad explicit immune exclusion; X-S, selective phenotype or threshold explicitly excluded; U, no major posted exclusion identified but actual enrollment unresolved; P, immune participants demonstrably enrolled but pooled; R, phenotype-resolved numbers and outcomes. These states address transportability only. Methodological credibility was considered separately.

Supplementary Table S2. Trial-level pivotal evidence map

Each protocol identifier occupies a separate row. Registry wording is quoted where concise and otherwise faithfully abstracted; “not stated” means that the posted summary reviewed did not provide the requested threshold. Eligibility without subgroup reporting is coded U rather than interpreted as positive direct evidence.

| **Agent/trial/registry** | **Syndrome/pathogen** | **Posted immune criterion** | **Corticosteroid dose/duration** | **Neutrophil threshold** | **Severity restriction** | **Immune-host eligibility** | **Demonstrably enrolled** | **Phenotype-specific numbers/outcomes** | **State and source** |
| --- | --- | --- | --- | --- | --- | --- | --- | --- | --- |
| Ceftazidime–avibactam \| RECLAIM 1 [NCT01499290](https://clinicaltrials.gov/study/NCT01499290) | cIAI; broad Gram-negative eligibility | No broad immune exclusion in posted record; high-mortality septic shock/terminal illness excluded. | None stated | None stated | High-mortality septic shock; unlikely survival through follow-up | Allowed or not expressly excluded; actual eligibility/enrollment unresolved | Not reported | No | U; ClinicalTrials.gov record (last posted 6 Sep 2017; paired publication: Mazuski et al., 2016) |
| Ceftazidime–avibactam \| RECLAIM 2 [NCT01500239](https://clinicaltrials.gov/study/NCT01500239) | cIAI; broad Gram-negative eligibility | Paired protocol now shown as an alias of the RECLAIM registry record; no broad immune exclusion identified. | None stated | None stated | High-mortality septic shock; unlikely survival through follow-up | Allowed or not expressly excluded; actual eligibility/enrollment unresolved | Not reported | No | U; ClinicalTrials.gov alias/current consolidated record (last posted 6 Sep 2017; Mazuski et al., 2016) |
| Ceftazidime–avibactam \| RECLAIM 3 [NCT01726023](https://clinicaltrials.gov/study/NCT01726023) | cIAI; Asian phase 3 population | No broad immune exclusion in posted record; high-mortality septic shock/terminal illness excluded. | None stated | None stated | High-mortality septic shock; unlikely survival through follow-up | Allowed or not expressly excluded; actual eligibility/enrollment unresolved | Not reported | No | U; ClinicalTrials.gov record (last posted 6 Sep 2017) |
| Ceftazidime–avibactam \| RECAPTURE 1 [NCT01595438](https://clinicaltrials.gov/study/NCT01595438) | cUTI/acute pyelonephritis | “Immunocompromised” and prior renal transplant excluded. | Not specified in posted summary | Not specified | High-mortality septic shock/terminal illness | No for at least one broad major phenotype | Excluded phenotype(s) not enrolled | No | X-B; ClinicalTrials.gov record (last posted 6 Sep 2017; Wagenlehner et al., 2016) |
| Ceftazidime–avibactam \| RECAPTURE 2 [NCT01599806](https://clinicaltrials.gov/study/NCT01599806) | cUTI/acute pyelonephritis | “Immunocompromised” and prior renal transplant excluded. | Not specified in posted summary | Not specified | High-mortality septic shock/terminal illness | No for at least one broad major phenotype | Excluded phenotype(s) not enrolled | No | X-B; ClinicalTrials.gov record (last posted 6 Sep 2017; Wagenlehner et al., 2016) |
| Ceftazidime–avibactam \| REPROVE [NCT01808092](https://clinicaltrials.gov/study/NCT01808092) | HAP/VAP; Gram-negative pathogens | No broad immune exclusion identified in the posted registry summary. | None stated | None stated | Protocol-defined severity restrictions; immune phenotype not a reported stratum | Allowed or not expressly excluded; actual eligibility/enrollment unresolved | Not reported | No | U; ClinicalTrials.gov record (last posted 6 Sep 2017; Torres et al., 2018) |
| Ceftazidime–avibactam \| REPRISE [NCT01644643](https://clinicaltrials.gov/study/NCT01644643) | cUTI/cIAI due to ceftazidime-resistant Enterobacterales or *P. aeruginosa* | “Immunocompromised” and prior renal transplant excluded. | Not specified in posted summary | Not specified | APACHE II >30 for cIAI; severe septic shock/high mortality risk | No for at least one broad major phenotype | Excluded phenotype(s) not enrolled | No | X-B; ClinicalTrials.gov record (last posted 29 Sep 2017; Carmeli et al., 2016) |
| Ceftolozane–tazobactam \| ASPECT-cUTI protocol A [NCT01345929](https://clinicaltrials.gov/study/NCT01345929) | cUTI/pyelonephritis | AIDS, hematological malignancy, bone-marrow transplant, cancer chemotherapy, anti-rejection therapy, and qualifying corticosteroids excluded. | Prednisone-equivalent ≥40 mg/day continuously for >14 days before randomization | ANC <500 cells/μL | Rapidly progressive/life-threatening illness, respiratory failure, or septic shock | No for at least one broad major phenotype | Excluded phenotype(s) not enrolled | No | X-B; ClinicalTrials.gov record (last posted 25 Oct 2018; Wagenlehner et al., 2015) |
| Ceftolozane–tazobactam \| ASPECT-cUTI protocol B [NCT01345955](https://clinicaltrials.gov/study/NCT01345955) | cUTI/pyelonephritis | Paired protocol now shown as a registry alias; same broad immune exclusions as protocol A. | Prednisone-equivalent ≥40 mg/day continuously for >14 days before randomization | ANC <500 cells/μL | Rapidly progressive/life-threatening illness, respiratory failure, or septic shock | No for at least one broad major phenotype | Excluded phenotype(s) not enrolled | No | X-B; ClinicalTrials.gov alias/current consolidated record (last posted 25 Oct 2018; Wagenlehner et al., 2015) |
| Ceftolozane–tazobactam \| ASPECT-cIAI protocol A [NCT01445665](https://clinicaltrials.gov/study/NCT01445665) | cIAI requiring source control | Paired protocol now shown as a registry alias; no broad immune exclusion identified. | None stated | None stated | Rapidly progressive/life-threatening illness, respiratory failure, or septic shock | Allowed or not expressly excluded; actual eligibility/enrollment unresolved | Not reported | No | U; ClinicalTrials.gov alias/current consolidated record (last posted 16 Nov 2018; Solomkin et al., 2015) |
| Ceftolozane–tazobactam \| ASPECT-cIAI protocol B [NCT01445678](https://clinicaltrials.gov/study/NCT01445678) | cIAI requiring source control | No broad immune exclusion identified in posted record. | None stated | None stated | Rapidly progressive/life-threatening illness, respiratory failure, or septic shock | Allowed or not expressly excluded; actual eligibility/enrollment unresolved | Not reported | No | U; ClinicalTrials.gov record (last posted 16 Nov 2018; Solomkin et al., 2015) |
| Ceftolozane–tazobactam \| ASPECT-NP [NCT02070757](https://clinicaltrials.gov/study/NCT02070757) | Ventilated HAP/VAP | Active immunosuppression excluded. | Not specified in posted summary | Not specified | Severe confounding respiratory conditions excluded | No for at least one broad major phenotype | Excluded phenotype(s) not enrolled | No | X-B; ClinicalTrials.gov record (last posted 5 May 2020; Kollef et al., 2019) |
| Meropenem–vaborbactam \| TANGO I [NCT02166476](https://clinicaltrials.gov/study/NCT02166476) | cUTI/acute pyelonephritis | Renal transplant, recent cancer chemotherapy/transplant immunosuppression, HIV with CD4 <200 cells/μL, hematological malignancy, bone-marrow transplant, and systemic corticosteroids excluded. | Long-term systemic corticosteroids for ≥2 weeks; dose not stated | ANC <1,000 cells/μL | Shock/profound hypotension and other severe-sepsis criteria | No for at least one broad major phenotype | Excluded phenotype(s) not enrolled | No | X-B; ClinicalTrials.gov record (last posted 11 Jun 2018; Kaye et al., 2018) |
| Meropenem–vaborbactam \| TANGO II [NCT02168946](https://clinicaltrials.gov/study/NCT02168946) | Serious CRE infections (cUTI, HAP/VAP, bacteremia, cIAI) | Current registry wording lists significant hematological or immunological disease/dysfunction as an exclusion; however, the published TANGO II report states that immunocompromised patients, including those with leukemia/lymphoma, prior solid-organ or stem-cell transplantation, neutropenia, or active immunosuppressive therapy, were eligible. | High-dose systemic corticosteroid therapy for ≥2 weeks was included within the definition of immunocompromise and was permitted. | Neutropenia was permitted; ANC <1,000 cells/mm³ was included within the immunocompromised definition. | APACHE II >30; expected survival ≤72 h | Yes; immunocompromised patients were explicitly eligible in the published trial. | Yes; immunocompromised patients were enrolled. | Pooled immunocompromised subgroup reported (11 meropenem–vaborbactam vs 8 BAT in the confirmed-CRE population; clinical cure at TOC 63.6% vs 0%); outcomes were not resolved separately by specific immune phenotype. | P; ClinicalTrials.gov record and published trial report (Wunderink et al., 2018). |
| Imipenem–cilastatin–relebactam \| RESTORE-IMI 1 [NCT02452047](https://clinicaltrials.gov/study/NCT02452047) | Imipenem-nonsusceptible HAP/VAP, cIAI, or cUTI | No broad immune exclusion identified in posted record. | None stated | None stated | Small pathogen-directed trial (50 participants); protocol severity restrictions | Allowed or not expressly excluded; actual eligibility/enrollment unresolved | Not reported | No | U; ClinicalTrials.gov record (last posted 19 Oct 2018; Motsch et al., 2020) |
| Imipenem–cilastatin–relebactam \| RESTORE-IMI 2 [NCT02493764](https://clinicaltrials.gov/study/NCT02493764) | HABP/VABP | Active immunosuppression—medication or immunodeficiency-associated condition—excluded. | Not separately stated | Not separately stated | Protocol-defined pneumonia/severity criteria | No for at least one broad major phenotype | Excluded phenotype(s) not enrolled | No | X-B; ClinicalTrials.gov record (last posted 16 Apr 2020; Titov et al., 2021) |
| Cefiderocol \| APEKS-cUTI [NCT02321800](https://clinicaltrials.gov/study/NCT02321800) | cUTI/pyelonephritis | No broad immune exclusion identified in posted record. | None stated | None stated | Protocol-defined renal/severity restrictions | Allowed or not expressly excluded; actual eligibility/enrollment unresolved | Not reported | No | U; ClinicalTrials.gov record (last posted 12 Dec 2019; Portsmouth et al., 2018) |
| Cefiderocol \| APEKS-NP [NCT03032380](https://clinicaltrials.gov/study/NCT03032380) | Gram-negative HAP/VAP/health-care-associated pneumonia | No broad immune exclusion identified in posted record. | None stated | No immune-related threshold stated | Protocol-defined pneumonia/severity restrictions | Allowed or not expressly excluded; actual eligibility/enrollment unresolved | Not reported | No | U; ClinicalTrials.gov record (last posted 13 Nov 2020; Wunderink et al., 2021) |
| Cefiderocol \| CREDIBLE-CR [NCT02714595](https://clinicaltrials.gov/study/NCT02714595) | Severe carbapenem-resistant Gram-negative infection | Selected high-risk states excluded rather than all immunocompromise. | None stated | PMN <100 cells/μL | Refractory septic shock; APACHE II >30 | Partly; selected phenotype/threshold excluded | Excluded subgroup not enrolled; other immune enrollment unreported | No | X-S; ClinicalTrials.gov record (last posted 12 Jan 2021; Bassetti et al., 2021) |
| Sulbactam–durlobactam \| ATTACK [NCT03894046](https://clinicaltrials.gov/study/NCT03894046) | *A. baumannii–calcoaceticus* complex HAP/VAP or bacteremia | No broad immune exclusion identified in posted record. | None stated | None stated | Required APACHE II 10–30 or SOFA 7–11; sustained shock excluded | Allowed or not expressly excluded; actual eligibility/enrollment unresolved | Not reported | No | U; ClinicalTrials.gov record (last posted 1 Feb 2023; Kaye et al., 2023) |
| Aztreonam–avibactam \| REVISIT [NCT03329092](https://clinicaltrials.gov/study/NCT03329092) | cIAI or HAP/VAP due to Gram-negative bacteria | Severe neutropenia and syndrome-specific prior transplants excluded. | None stated | ANC <500 cells/μL | APACHE II >30; APACHE II <10; transplant restrictions varied by syndrome | Partly; selected phenotype/threshold excluded | Excluded subgroup not enrolled; other immune enrollment unreported | No | X-S; ClinicalTrials.gov record (last posted 10 Dec 2024; Carmeli et al., 2025) |
| Aztreonam–avibactam \| ASSEMBLE [NCT03580044](https://clinicaltrials.gov/study/NCT03580044) | Serious MBL-producing Gram-negative infection | No broad immune exclusion identified in posted record. | None stated | No immune-related threshold stated | Single-arm cohort of 15; syndrome-specific severity criteria | Allowed or not expressly excluded; actual eligibility/enrollment unresolved | Not reported | No | U; ClinicalTrials.gov record (last posted 2 Feb 2024; Carmeli et al., 2025) |
| Cefepime–enmetazobactam \| ALLIUM [NCT03687255](https://clinicaltrials.gov/study/NCT03687255) | cUTI/pyelonephritis | AIDS/CD4 <200 cells/μL, hematological malignancy, bone-marrow transplant, chemotherapy, anti-rejection therapy, and qualifying corticosteroids excluded. | Prednisone-equivalent ≥20 mg/day continuously for >14 days before randomization | ANC <1,000 cells/μL | Life-threatening illness or organ-dysfunction sepsis | No for at least one broad major phenotype | Excluded phenotype(s) not enrolled | No | X-B; ClinicalTrials.gov record (last posted 10 Mar 2020; Kaye et al., 2022) |
| Plazomicin \| EPIC [NCT02486627](https://clinicaltrials.gov/study/NCT02486627) | cUTI/acute pyelonephritis | Documented immunodeficiency or immunocompromised condition excluded. | Not specified | Not specified | Rapidly progressing or immediately life-threatening illness | No for at least one broad major phenotype | Excluded phenotype(s) not enrolled | No | X-B; ClinicalTrials.gov record (last posted 23 Aug 2018; Wagenlehner et al., 2019) |
| Plazomicin \| CARE [NCT01970371](https://clinicaltrials.gov/study/NCT01970371) | CRE bacteremia, HAP/VAP, cUTI, or acute pyelonephritis | Renal transplant excluded for the cUTI/pyelonephritis stratum; no broad immune exclusion stated for all syndromes. | None stated | None stated | APACHE II 15–30 in randomized cohort; ≤30 in expansion cohort | Partly; selected phenotype/threshold excluded | Excluded subgroup not enrolled; other immune enrollment unreported | No | X-S; ClinicalTrials.gov record (last posted 16 Oct 2018; McKinnell et al., 2019) |
| Eravacycline \| IGNITE1 [NCT01844856](https://clinicaltrials.gov/study/NCT01844856) | cIAI | HIV requiring therapy or CD4 <300 cells/μL, transplant, hematological malignancy, and recent antineoplastic therapy excluded. | Prednisone-equivalent >40 mg/day for >2 weeks | Not specified | Protocol-defined surgical and severity exclusions | No for at least one broad major phenotype | Excluded phenotype(s) not enrolled | No | X-B; ClinicalTrials.gov record (last posted 11 Jan 2022; Solomkin et al., 2017) |
| Eravacycline \| IGNITE4 [NCT02784704](https://clinicaltrials.gov/study/NCT02784704) | cIAI | HIV, transplant, hematological malignancy, and recent antineoplastic therapy excluded. | Not specified in posted summary | Not specified | Protocol-defined surgical and severity exclusions | No for at least one broad major phenotype | Excluded phenotype(s) not enrolled | No | X-B; ClinicalTrials.gov record (last posted 6 Jan 2022; Solomkin et al., 2019) |

**Core-map summary: At the protocol-identifier level, 15/27 protocols (56%) explicitly excluded at least one major immune phenotype or threshold, including 12 classified as X-B and three as X-S; 11/27 protocols (41%) were classified as U, one of 27 (4%) was classified as P, and 0/27 reported comparative outcomes resolved to a defined immune phenotype. Three paired protocols are represented in the current registry by a primary record plus an alias. After exclusion of these aliases, 14/24 distinct current registry records (58%) were classified as X-B or X-S, nine of 24 (38%) were classified as U, one of 24 (4%) was classified as P, and none reported comparative outcomes resolved to a defined immune phenotype.**

Supplementary Table S3. Immune-host post-approval cohort map

CAZ–AVI, ceftazidime–avibactam; C/T, ceftolozane–tazobactam; BAT, best available therapy; SOT, solid-organ transplantation; HCT, hematopoietic cell transplantation. A blank variable was not assumed to be absent; incompletely reported variables are labeled explicitly.

| **Study / design** | **Immune phenotype / definition / N** | **Pathogen, mechanism, source, bacteremia** | **Intervention, comparator, empirical/definitive use, time zero** | **Severity, source control, immune recovery, co-therapy** | **Adjustment / covariates** | **Clinical / microbiological outcomes** | **Major bias concerns** | **Transportability / credibility** |
| --- | --- | --- | --- | --- | --- | --- | --- | --- |
| Abi Frem et al., 2024; retrospective case–control | Acute leukemia after induction or HCT with febrile neutropenia; 339 episodes (39 CAZ–AVI, 300 BAT) | Mixed suspected/documented infection; Gram-negative organisms enriched in CAZ–AVI arm; device and respiratory sources common | CAZ–AVI versus best available therapy; CAZ–AVI commonly used after failure/escalation, so treatment time zero was not aligned | CAZ–AVI arm had more documented infection/sepsis; source control, ANC recovery, and co-therapy were not consistently standardized. | Backward logistic regression including severity indices; organism, source, and treatment timing remained incompletely controlled | Mortality 23.1% versus 3.0%; adjusted association favored BAT, interpreted as strong confounding by indication rather than a causal drug effect | Severe channeling, calendar/access effects, episode-level reuse, residual confounding, survivor/immortal-time risk | Phenotype-specific comparative; high transportability, low methodological credibility |
| Bergas et al., 2022 (ZENITH); matched multicenter cohort | Neutropenic hematology patients; 44 C/T cases and 88 matched controls | *P. aeruginosa* bloodstream infection; 91% MDR; endogenous and pulmonary sources prominent | C/T versus other active regimens; empirical and definitive use mixed | Pneumonia, profound neutropenia, and persistent bacteremia considered; source control and exposure remained incompletely captured. | Matching plus multivariable adjustment; pneumonia, profound neutropenia, persistent bacteremia considered | 7-day mortality 6.8% versus 34.1%; 30-day mortality 22.7% versus 48.9%; adjusted OR for death 0.19 | Nonrandom treatment choice, calendar-era and susceptibility differences, unmeasured source control/exposure | Phenotype-specific comparative; moderate credibility, high transportability |
| Copaja-Corzo et al., 2025; retrospective cohort | Acute leukemia; 41 patients, 92.7% neutropenic at bloodstream-infection onset | Carbapenemase-producing Enterobacterales bloodstream infection | Targeted CAZ–AVI in approximately half versus other therapy (often colistin); median CAZ–AVI initiation 8 days | Severity modeled; 92.7% neutropenic; source control, neutrophil recovery, and co-therapy incompletely reported. | Cox regression; severity and treatment variables included but timing alignment limited | 30-day mortality 60.9%; CAZ–AVI associated with lower mortality (adjusted HR 0.29, 95% CI 0.09–0.92) | Small sample, delayed exposure, confounding by indication, immortal-time risk, imprecision | Phenotype-specific comparative; moderate-to-low credibility, high transportability |
| Halacoglu et al., 2025; single-center retrospective series | Hematological malignancy/HCT; 80 CAZ–AVI-treated patients; 52.5% had ANC <100 cells/μL at initiation | Resistant Gram-negative infections; pneumonia and mechanical ventilation prominent among deaths | CAZ–AVI; no concurrent comparator; empirical/definitive timing heterogeneous | ANC <100 cells/μL, pneumonia, ventilation, and neutropenia duration reported; source control and co-therapy heterogeneous. | Multivariable prognostic analysis, not a treatment-effect model | 28-day mortality 57.5%; pneumonia/mechanical ventilation and duration of neutropenia independently associated with death | No comparator, heterogeneous indications, severity and salvage-treatment selection | Phenotype-specific descriptive; low treatment-effect credibility, high transportability |
| Hart et al., 2021; 14-center retrospective series | Broad immunocompromise; 69 patients (68% solid-organ transplant; 84% receiving immunosuppressants) | MDR *P. aeruginosa;* respiratory source 56%, wound 11%; bacteremia variably present | C/T; no concurrent comparator; dose varied, including pneumonia and lower regimens | Respiratory source and immunosuppression reported; dose varied; source control and immune trajectory incompletely reported. | Descriptive outcomes and exploratory classification-tree analysis | Clinical cure 68%; 30-day mortality 19%; pneumonia cure 75% with pneumonia dose versus 30% with lower dose | Dose confounding, no comparator, mixed phenotypes/sources, small subgroups | Heterogeneous immune cohort; moderate transportability for SOT, low causal credibility |
| Herrera et al., 2025; prospective cohort | Immunosuppressed patients; 82 infection episodes (hematological malignancy and SOT predominant) | Carbapenemase-producing Enterobacterales; KPC most common, followed by MBL; 44 bacteremic and 38 non-bacteremic | CAZ–AVI or CAZ–AVI plus aztreonam selected by mechanism; definitive treatment ≥72 h | Shock, refractory malignancy, bacteremia, and mechanism-directed co-therapy reported; source control and immune recovery incomplete. | Propensity-score-adjusted logistic regression comparing bacteremic with non-bacteremic episodes | 7-day response 88.6%–92.1%; 30-day mortality 27.3% in bacteremia and 10.5% without bacteremia; shock and refractory malignancy predicted death | No randomized regimen comparison, mechanism-driven channeling, 72-h survivor requirement | Phenotype-specific descriptive/comparative by syndrome; moderate transportability, low regimen-effect credibility |
| Lombardi et al., 2025; prospective multicenter cohort analysis | 84 immunocompromised among 185 cefiderocol-treated adults; immune phenotypes pooled | MDR Gram-negative infections; Enterobacterales, *P. aeruginosa*, and *A. baumannii*; sources heterogeneous | Cefiderocol; immunocompromised versus non-immunocompromised comparison; empirical/targeted and combination/monotherapy mixed | Pathogen and clinical factors considered; phenotype, sources, empirical/targeted use, and co-therapy pooled. | Cox regression for mortality; organism and clinical factors considered | 30-day mortality 40.8% versus 33.3% (P=0.543); targeted 28-day cure 77.3% Enterobacterales, 81% *P. aeruginosa*, 42% *A. baumannii* | Phenotype pooling, confounding by pathogen/severity, no active-drug comparator | Immune-host representation confirmed but pooled; moderate credibility for prognosis, low for comparative effectiveness |
| Sastre-Escolà et al., 2025 (TARZAN); multicenter retrospective series | Neutropenic onco-hematology patients; 54 episodes | Carbapenemase-producing Enterobacterales bloodstream infection | Targeted CAZ–AVI in all episodes; 30% empirical; 55.5% definitive combination therapy | Shock, initial activity, and combination therapy reported; source control and neutrophil recovery not uniformly resolved. | Multivariable prognostic analysis; no concurrent treatment comparator | 7-day and 30-day mortality 11% and 24%; septic shock independently predicted 30-day death; 47% initial empirical therapy inadequate | Selected survivors receiving CAZ–AVI, combination confounding, no comparator, limited power | Phenotype-specific descriptive; high transportability, low treatment-effect credibility |
| Tumbarello et al., 2025; 17-center retrospective cohort | Hematological malignancy; 198 patients | 66 fever of unknown origin and 132 microbiologically proven infections; KPC Enterobacterales and resistant *P. aeruginosa* prominent | CAZ–AVI for suspected or proven infection; no active concurrent comparator | Shock and initial active therapy reported; source control and longitudinal neutrophil recovery incompletely reported. | Multivariable prognostic analysis including septic shock and appropriateness of initial therapy | 30-day mortality 17.7%; shock and inappropriate initial therapy independently predicted death; four relapses | Mixed suspected/proven infection, no comparator, center and treatment-timing heterogeneity | Phenotype-specific descriptive; high transportability, low treatment-effect credibility |
| Zhang et al., 2024; single-center retrospective cohort | Kidney-transplant recipients; 81 patients | Carbapenem-resistant Gram-negative infection (61 CRKP, 16 CRPA, 4 CRAB); sources heterogeneous | CAZ–AVI; monotherapy versus combination explored; treatment within 48 h assessed | APACHE II, timing, and mono/combination use reported; sources heterogeneous and source control not standardized. | Multivariable logistic regression for mortality and cure | 30-day mortality 22.2%; clinical cure 72.8%; microbiological cure 66.7%; APACHE II predicted death | No untreated concurrent comparator, source and co-therapy confounding, imprecise timing estimates | Phenotype-specific descriptive; high SOT transportability, low comparative-effect credibility |
| Zhang et al., 2026; propensity-matched retrospective cohort | Liver-transplant recipients; 45 treated, 44 matched (22 per group) | Postoperative carbapenem-resistant organism infection, predominantly carbapenem-resistant *K. pneumoniae* | CAZ–AVI versus polymyxin B as primary regimen; required survival ≥72 h after initiation | Postoperative setting, severity matching, and nephrotoxicity considered; survival ≥72 h required; source control incomplete. | 1:1 propensity-score matching; residual severity and nephrotoxin confounding remained | No clear efficacy difference in the small matched sample; acute kidney injury was more frequent with polymyxin B | Single center, small sample, survivor selection, incomplete culture follow-up, residual confounding | Phenotype-specific comparative; moderate transportability, low-to-moderate credibility |

Methodological credibility was assessed qualitatively rather than through a numerical risk-of-bias score. The appraisal considered treatment-time-zero alignment, availability and comparability of a concurrent control group, confounding by indication, adjustment for baseline severity and infection source, timing of active therapy, source control, immune recovery, dosing and co-therapy, outcome ascertainment, sample size, and statistical precision. These labels summarize treatment-effect interpretability and should not be interpreted as validated certainty-of-evidence grades. Phenotype-specific transportability was assessed separately.

Supplementary Table S4. Phenotype-by-agent evidence-gap matrix

Codes summarize the best phenotype-specific post-approval evidence identified: C, comparative cohort; D, descriptive phenotype-specific cohort/series; H, heterogeneous pooled immune cohort; PK, pediatric pharmacokinetic/safety evidence without phenotype-specific treatment-effect evidence; —, no core phenotype-specific treatment-outcome evidence identified. These codes are not certainty grades.

| **Target phenotype** | **CAZ–AVI / related CRE options** | **C/T / antipseudomonal options** | **Meropenem–vaborbactam or imipenem–relebactam** | **Cefiderocol** | **Sulbactam–durlobactam** | **Aztreonam protected by avibactam** | **Cefepime–enmetazobactam / plazomicin / eravacycline** |
| --- | --- | --- | --- | --- | --- | --- | --- |
| Hematological malignancy, profound neutropenia, or HCT | C/D: several comparative/descriptive cohorts | C: ZENITH; D: additional series | — | H: pooled immune cohort | — | D: CAZ–AVI + aztreonam in mixed cohort | — |
| Solid-organ transplantation | C/D: kidney and liver transplant cohorts | H: SOT majority in mixed cohort | — | H: pooled immune cohort | — | D: mixed SOT/hematology cohort | — |
| Solid tumor receiving cytotoxic chemotherapy | H: usually pooled or undefined | H: broad immune cohorts only | — | H: pooled immune cohort | — | — | — |
| CAR-T/cell therapy or B-cell depletion | — | — | — | — | — | — | — |
| Autoimmune/inflammatory disease on intensive therapy | H: broad immune cohorts only | H: broad immune cohorts only | — | H: pooled immune cohort | — | — | — |
| Advanced HIV infection or inborn error of immunity | — | — | — | — | — | — | — |
| Pediatric patient within any phenotype (cross-cutting modifier) | PK/safety and non-immune pediatric trials | PK/safety and non-immune pediatric trials | — | PK/safety; immune-host outcomes absent | — | — | — |

Supplementary Table S5. Structured methodological limitations of the 11 phenotype-specific treatment cohorts

| **Study/design** | **Selection/treatment channeling** | **Treatment time-zero alignment** | **Control of measured confounding** | **Source control, co-therapy, and immune recovery** | **Outcome ascertainment** | **Statistical precision** | **Principal interpretive limitation** |
| --- | --- | --- | --- | --- | --- | --- | --- |
| Abi Frem et al., 2024; retrospective case–control | Severe treatment channeling; CAZ–AVI preferentially used after failure/escalation; calendar/access effects and episode-level reuse | Not aligned; CAZ–AVI frequently initiated after treatment failure/escalation | Backward logistic regression including severity indices; organism, source, and treatment timing remained incompletely controlled | Source control, ANC recovery, and co-therapy not consistently standardized | Retrospective; mortality objectively ascertainable, but treatment-effect attribution limited | Limited for CAZ–AVI exposure; 39 CAZ–AVI episodes among 339 episodes | Apparent treatment differences are highly vulnerable to confounding by indication, survivor/immortal-time bias, and unequal treatment timing and should not be interpreted causally |
| Bergas et al., 2022 (ZENITH); matched multicenter cohort | Nonrandom treatment selection; calendar-era and susceptibility differences remain possible | Partially aligned; empirical and definitive treatment use were mixed | Matching plus multivariable adjustment incorporating pneumonia, profound neutropenia, and persistent bacteremia | Source control and antibacterial exposure incompletely captured; relevant neutropenia/severity variables considered | Retrospective; short- and longer-term mortality clearly reported | Limited-to-moderate; 44 C/T cases and 88 matched controls | Provides one of the stronger phenotype-specific comparative signals, but residual treatment selection, calendar-era effects, and unmeasured source control/exposure prevent causal equivalence to randomized evidence |
| Copaja-Corzo et al., 2025; retrospective cohort | Nonrandom treatment allocation with likely confounding by indication; delayed CAZ–AVI exposure | Poorly aligned; median CAZ–AVI initiation approximately 8 days after infection onset, creating immortal-time risk | Cox regression incorporating severity and treatment variables; treatment-timing alignment remained limited | Source control, neutrophil recovery, and co-therapy incompletely reported | Retrospective; 30-day mortality objectively defined | Limited; 41 patients and wide treatment-effect confidence interval | The association between CAZ–AVI and lower mortality is vulnerable to delayed-exposure and immortal-time bias, residual confounding, and substantial imprecision |
| Halacoglu et al., 2025; single-center retrospective series | Treated-only cohort; heterogeneous indications and possible salvage-treatment selection | Heterogeneous; empirical/definitive timing varied and no concurrent comparator was available | Multivariable prognostic analysis; not designed as a treatment-effect model | ANC, pneumonia, ventilation and duration of neutropenia reported; source control and co-therapy heterogeneous | Retrospective; 28-day mortality clearly ascertainable | Moderate for prognostic description (80 patients), but insufficient for comparative treatment effects | Useful for identifying prognostic factors in profoundly immunocompromised patients receiving CAZ–AVI, but cannot establish drug effectiveness relative to alternative regimens |
| Hart et al., 2021; 14-center retrospective series | Treated-only cohort with heterogeneous immune phenotypes, infection sources, and dosing; potential dose-by-indication channeling | No comparative time-zero alignment; no concurrent comparator and dosing/treatment context varied | Descriptive analyses and exploratory classification-tree analysis; no comparative treatment adjustment | Immunosuppression and respiratory source reported; dosing varied; source control and immune trajectory incompletely characterized | Retrospective; clinical cure and 30-day mortality reported, with heterogeneous clinical contexts | Limited; 69 patients with small dose- and syndrome-specific subgroups | Supports real-world C/T experience, particularly in SOT, but dose comparisons and outcome differences are strongly confounded by phenotype, source, disease severity, and treatment selection |
| Herrera et al., 2025; prospective cohort | Mechanism-directed treatment selection; inclusion required ≥72 h of definitive treatment, creating survivor selection | Partially aligned; definitive therapy was defined, but the ≥72-h treatment requirement conditions inclusion on survival after treatment initiation | Propensity-score-adjusted logistic regression comparing bacteremic and non-bacteremic episodes; not a randomized regimen comparison | Shock, refractory malignancy, bacteremia and mechanism-directed co-therapy reported; source control and immune recovery incompletely characterized | Prospective assessment of early response and 30-day mortality | Moderate for prognostic associations (82 episodes), limited for individual regimen effects | Supports phenotype-specific outcome description and mechanism-directed treatment, but cannot isolate the effect of individual regimens because of mechanism-driven channeling and the ≥72-h survivor requirement |
| Lombardi et al., 2025; prospective multicenter cohort analysis | Immunocompromised patients embedded within a broader cefiderocol-treated population; phenotypes and treatment contexts pooled | Partially aligned / heterogeneous; empirical versus targeted and monotherapy versus combination therapy were mixed | Cox regression for mortality including organism and clinical factors; no active-drug treatment comparator | Infection sources, immune phenotypes, empirical/targeted therapy and co-therapy pooled; source control and immune trajectory not phenotype-resolved | Prospective mortality and cure assessment | Moderate for prognostic description (84 immunocompromised among 185 adults), but limited for phenotype-specific comparative effectiveness | Confirms cefiderocol use in immunocompromised hosts but pooled immune phenotypes, pathogen/severity confounding, and absence of an active comparator prevent phenotype-specific treatment-effect inference |
| Sastre-Escolà et al., 2025 (TARZAN); multicenter retrospective series | Selected CAZ–AVI-treated cohort; possible survivor selection and substantial combination-therapy confounding | Treatment initiation was identifiable within the treated cohort, but no comparative time-zero alignment was possible | Multivariable prognostic analysis; no concurrent treatment comparator | Shock, initial treatment activity and combination therapy reported; source control and neutrophil recovery not uniformly resolved | Retrospective; 7- and 30-day mortality objectively reported | Limited; 54 episodes | Highly relevant to neutropenic onco-hematology patients, but provides descriptive/prognostic rather than comparative effectiveness evidence because all patients ultimately received CAZ–AVI |
| Tumbarello et al., 2025; 17-center retrospective cohort | Treated-only cohort including suspected and microbiologically proven infection; center and treatment-timing heterogeneity | Heterogeneous; no active concurrent comparator and timing of CAZ–AVI use varied | Multivariable prognostic analysis including septic shock and appropriateness of initial therapy | Initial active therapy reported; source control and longitudinal neutrophil recovery incompletely characterized | Retrospective; 30-day mortality and relapse reported | Relatively stronger for prognosis (198 patients), but comparative treatment-effect precision cannot be estimated without a comparator | Large phenotype-specific experience supports prognostic interpretation and importance of early active therapy, but does not establish the comparative effectiveness of CAZ–AVI |
| Zhang et al., 2024; single-center retrospective cohort | CAZ–AVI-treated kidney-transplant cohort; no untreated or alternative-active concurrent comparator; mono/combination treatment selection may be confounded | Partially characterized; treatment within 48 h was assessed, but comparative alignment was not possible | Multivariable logistic regression for mortality and clinical cure | APACHE II, treatment timing and mono/combination use reported; infection sources heterogeneous and source control not standardized | Retrospective clinical and microbiological outcome assessment | Moderate for descriptive/prognostic outcomes (81 patients), limited for comparative effects | Provides direct kidney-transplant experience and supports the importance of treatment timing, but cannot determine the causal effect of CAZ–AVI or combination therapy because of absent concurrent comparison and residual source/co-therapy confounding |
| Zhang et al., 2026; propensity-matched retrospective cohort | Single-center postoperative liver-transplant cohort; eligibility required survival ≥72 h after treatment initiation | Treatment initiation defined, but ≥72-h survivor requirement introduces survivor selection | 1:1 propensity-score matching; residual severity and nephrotoxin confounding remained | Postoperative context and nephrotoxicity considered; source control incomplete and nephrotoxic co-exposures remained potential confounders | Retrospective efficacy and safety assessment; culture follow-up incomplete | Very limited; 44 matched patients, 22 per treatment group | The comparative nephrotoxicity signal is more interpretable than the efficacy comparison; small sample size, survivor selection, incomplete microbiological follow-up, and residual confounding limit conclusions about comparative effectiveness |

ANC, absolute neutrophil count; CAZ–AVI, ceftazidime–avibactam; C/T, ceftolozane–tazobactam; HCT, hematopoietic cell transplantation; SOT, solid-organ transplantation. Methodological limitations were assessed descriptively across structured domains relevant to observational treatment studies: selection and treatment channeling, treatment time-zero alignment, control of measured confounding, reporting of source control/co-therapy/immune recovery, outcome ascertainment, and statistical precision. This domain-based appraisal was used to support interpretation of treatment-effect credibility and is not a validated risk-of-bias instrument. No numerical score or overall certainty-of-evidence grade was assigned. Phenotype-specific transportability was assessed separately in Supplementary Table S3.
